# The RNA degradation enzyme RNase E is essential for early flagellar assembly in *Escherichia coli*

**DOI:** 10.1093/pnasnexus/pgaf269

**Published:** 2025-08-18

**Authors:** Wei-Syuan Wang, Yu-Hsiang Chen, Gunn-Guang Liou, Oleg N Murashko, Sue Lin-Chao

**Affiliations:** Institute of Molecular Biology, Academia Sinica, Taipei 11529, Taiwan; Molecular and Cell Biology, Taiwan International Graduate Program, Academia Sinica and Graduate Institute of Life Science, National Defense Medical Center, Taipei 11490, Taiwan; Institute of Molecular Biology, Academia Sinica, Taipei 11529, Taiwan; Institute of Molecular Biology, Academia Sinica, Taipei 11529, Taiwan; Institute of Molecular Biology, Academia Sinica, Taipei 11529, Taiwan; Institute of Molecular Biology, Academia Sinica, Taipei 11529, Taiwan; Molecular and Cell Biology, Taiwan International Graduate Program, Academia Sinica and Graduate Institute of Life Science, National Defense Medical Center, Taipei 11490, Taiwan

**Keywords:** RNase E, posttranscriptional regulation, sRNAs, flagella, TLR5-NF-κB signaling

## Abstract

*Escherichia coli* endoribonuclease E (RNase E), encoded by the essential *rne* gene and conserved across γ-Proteobacteria, plays a central role in RNA processing and decay. We show here that *rne*-null strain, *rne*-null strain complemented with catalytic-null RNase E mutant, and C-terminal-truncated strain (Rned500) all lack flagellar biogenesis and motility under both aerobic and anaerobic conditions, which are restored by wild-type RNase E complementation. The Rned500 displays dysregulated expression of the three-tiered flagellar transcriptional cascade, increased stability of flagellar mRNAs, and reduced flagellar protein levels through sRNA-dependent translational inhibition. However, ectopic expression of flagellar master regulators or flagellar proteins fails to restore flagellar biogenesis and motility. To investigate the underlying defect, we examined the cellular localization of the early flagellar structural protein FliF and found it mislocalized in Rned500, indicating a disruption of early flagellar assembly. This defect is further supported by the impaired secretion of the flagellar anti-sigma factor FlgM in Rned500, a process that requires a functional flagellar basal body. Complementation with wild-type RNase E in Rned500 fully restores expression of the flagellar cascade, proper membrane localization of FliF, flagella formation, and motility. Wild-type RNase E–expressing strains, but not Rned500, activate Toll-like receptor 5 (TLR5)-dependent nuclear factor-kappa B signaling in THP-1 human monocytic cells through flagellin. This response, confirmed by a TLR5 dual-luciferase reporter assay in transfected HEK293T human embryonic kidney cells, highlights RNase E's role in enabling flagellar expression required for cellular immune activation. Collectively, these results identify RNase E as a key flagellar biogenesis regulator, revealing novel posttranscriptional control mechanisms.

Significance Statement RNase E catalytic activity is vital for cell viability. This study reveals a previously unrecognized role for RNase E and its catalytic activity in controlling the assembly and function of *Escherichia coli* flagella, which are essential for bacterial movement and immune system recognition. Using *rne* mutant strains, we show that RNase E is critical for proper flagellar gene expression, localization of flagellar structural protein for assembly, and for enabling flagellin-dependent immune responses in human cells. These findings establish a novel link between the RNA processing enzyme RNase E and bacterial motility, providing potential implications for understanding host–pathogen interactions.

## Introduction

*Escherichia coli* endoribonuclease E (RNase E), encoded by *rne* gene, is a single-stranded RNA endoribonuclease essential for cell viability (1–4). Temperature-sensitive *rne* strains exhibit defects in RNA processing and degradation at non-permissive temperatures (1, 4). Additionally, its cleavage activity controls ColE1 plasmid replication (5). RNase E comprises a highly conserved N-terminal catalytic domain and a less conserved C-terminal region (6, 7). The C-terminal region interacts with the DEAD-box RNA helicase RhlB, glycolytic enzyme enolase, and 3′ to 5′ exonuclease PNPase, forming the RNA degradosome (8, 9). RNase E participates in the maturation and degradation of all RNA species, including tRNAs, rRNAs, mRNAs, and sRNAs (reviewed in (10)). In C-terminal-truncated mutants such as rne131 and Rned500, catalytic activity is impaired, leading to RNA stabilization (11, 12). RNase E cleavage is often the rate-limiting step in RNA substrate degradation (13, 14), highlighting its critical role in posttranscriptional gene expression. While *rne* deletion is lethal, cell viability can be complemented by overexpression of the *rne* homolog *rng* (e.g. in strain KSL2004) (15). However, RNase G cannot fully substitute for RNase E's processing and degradation functions, underscoring the indispensability of RNase E in posttranscriptional regulation (15). Similarly, in *rne* deletion strain SK9714, which is complemented by *rne* overexpression, cell viability cannot be maintained through plasmid displacement with a plasmid carrying RNase E catalytic-null mutants (D303C, D346C, or both) (16, 17).

Flagella, essential for flagella-mediated motility, benefit bacteria in nutrient acquisition and niche colonization. In Gram-negative γ-Proteobacteria *E. coli* and *Salmonella*, flagellar biogenesis involves ∼50 genes whose expression is organized into a three-tiered transcriptional cascade (class I to class III), with transcription of lower-level genes dependent on the expression of higher-level genes (18). Apart from transcriptional regulation, flagellar genes are also posttranscriptionally regulated. In *E. coli*, CsrA has been shown to protect class I *flhDC* mRNA, which encodes the flagellar master regulators FlhDC, from RNase E–mediated cleavage, thereby activating its expression (19). Similarly, in *Salmonella*, deletion of *rraA*, encoding an RNase E repressor (20), leads to a reduction in the abundance of several flagellar class II mRNAs, including *fliDST*, which was demonstrated to be destabilized (21). Furthermore, a *Salmonella rne* C-terminal-truncated mutant (*rne*-*537*) exhibits reduced motility (22). Collectively, these studies suggest that RNase E regulation plays a crucial role in flagellar gene expression and motility in both *E. coli* and *Salmonella*. However, the fundamental role of RNase E in flagellar gene expression, biogenesis, and motility remains to be uncovered. Moreover, bacterial flagella can activate host innate immune responses through their flagellin (FliC) component via Toll-like receptor 5 (TLR5) and the downstream nuclear factor-kappa B (NF-κB) signaling pathway during bacteria–host interactions (23, 24). Whether RNase E is essential for triggering this signal transduction is unknown.

In this study, we investigate the role of RNase E in flagellar biogenesis, bacterial motility, and the cell immune response using genetic, transcriptomic, molecular, and biochemical analyses, along with motility assays, transmission electron microscopy (TEM), fluorescence imaging, and bacterial–cell interaction approaches. We demonstrate that RNase E is essential for flagellar biogenesis, motility, and flagellin-mediated activation of TLR5-dependent NF-κB signaling in human cell lines. These findings establish RNase E as a critical posttranscriptional regulator of bacterial flagellar biogenesis and motility, providing new insights into its broader regulatory functions.

## Results

### RNase E, but not RNase G, is crucial for flagella formation and bacterial motility

**Fig. 1.**
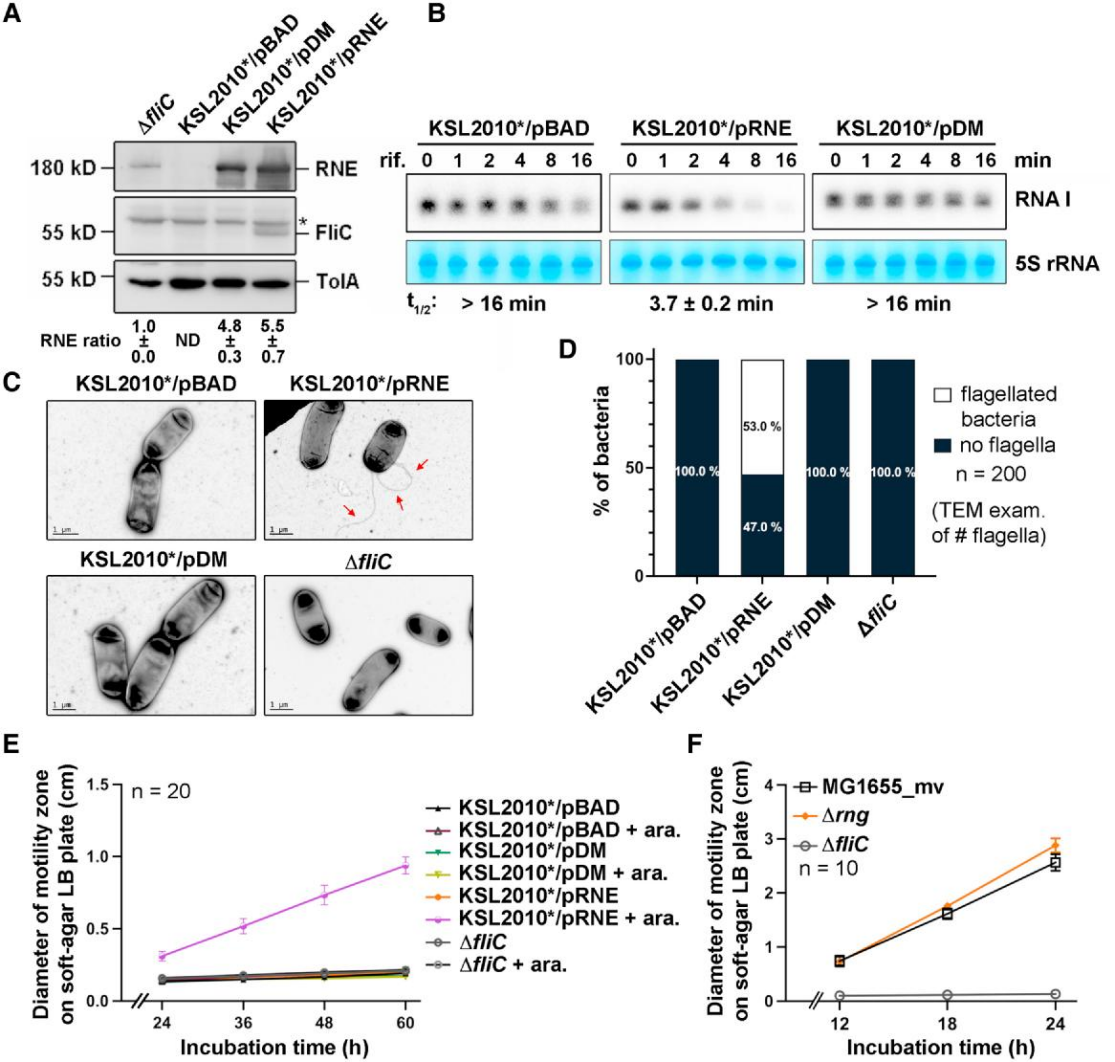
RNase E and its catalytic activity, but not RNase G, are essential for FliC expression, flagella formation, and bacterial motility. A) Western blot of RNase E, FliC, and TolA (loading control) in KSL2010*/pBAD, KSL2010*/pRNE, KSL2010*/pDM, and MG1655_mvΔ*fliC*. The MG1655_mvΔ*fliC* served as a FliC negative control and an RNase E–positive control. Antibodies against RNase E, FliC, and TolA were used. Asterisk, the nonspecific binding signal of FliC antibody. Protein markers are indicated in kD. Relative RNase E abundances are shown. Values are mean ± SD (*n* = 3 replicates). ND, not detected. B) Northern blot of RNA I abundance over time following rifampicin (rif.) treatment in KSL2010*/pBAD, KSL2010*/pRNE, and KSL2010*/pDM (*n* = 5 biological replicates). Methylene blue staining of 5S rRNA served as loading control. Half-lives (*t*_1/2_) are shown in minutes (m). Values are mean ± SD. C, D) Representative TEM images (C) and quantification of flagellated/nonflagellated cell percentage (D) from TEM images of KSL2010*/pBAD, KSL2010*/pRNE, KSL2010*/pDM, and MG1655_mvΔ*fliC* cells. Red arrows, flagellar filaments. Scale bar, 1 μm (*n* = 200 cells/strain). E) Motility zone diameters of KSL2010*/pBAD, KSL2010*/pRNE, KSL2010*/pDM, and MG1655_mvΔ*fliC* grown on LB soft-agar plates supplemented with or without 0.1% arabinose (ara.) for 60 hours (*n* = 20 biological replicates). Values are mean ± SD. F) Motility zone diameters of MG1655_mv, MG1655_mvΔ*rng*, and MG1655_mvΔ*fliC* grown on LB soft-agar plates for 24 h (*n* = 10 biological replicates). Values are mean ± SD.

To investigate the role of RNase E and its catalytic function in flagellar biogenesis and bacterial motility, we introduced plasmids expressing either wild-type RNase E (pRNE) or a catalytic-null D303N/D346N mutant (pDM) into the *rne*-null strain KSL2010*, which is supplemented with RNase G overexpression for cell viability (15). This generated KSL2010*/pRNE and KSL2010*/pDM strains. The D303N/D346N mutant produces full-length RNase E lacking catalytic activity (16, 17). Western blot confirmed similar levels of ectopically expressed wild-type and catalytic-null RNase E, both induced by 0.1% arabinose, in the chromosomally *rne*-null KSL2010*/pRNE and KSL2010*/pDM strains (Fig. 1A). The catalytic activity of RNase E was evaluated by measuring the degradation rate (half-life) of RNA I, a well-characterized RNase E substrate (3, 5, 25). Northern blot analysis showed that RNA I was rapidly degraded in KSL2010*/pRNE, with a half-life of 3.7 ± 0.2 min, whereas it remained stable in KSL2010*/pDM and the empty vector control strain, KSL2010*/pBAD, with half-lives exceeding 16 min (Fig. 1B), confirming that the catalytic-null D303N/D346N mutant lacks catalytic activity.

Using this established arabinose-inducible RNE variant expression system in *rne*-null strains, we examined the impact of RNase E expression and its catalytic activity on flagellar protein expression by analyzing the abundance of class III FliC, the major flagellar filament component (26). Western blot showed FliC expression exclusively in KSL2010*/pRNE, but not in KSL2010*/pBAD, KSL2010*/pDM, and Δ*fliC* (negative control) strains (Fig. 1A). TEM analysis revealed that 53% of KSL2010*/pRNE cells were flagellated, whereas all KSL2010*/pBAD, KSL2010*/pDM, and Δ*fliC* cells were nonflagellated (Fig. 1C and D). Moreover, motility assays, which measured the diameter of the motility zone for bacteria cultured on LB soft-agar plates, showed that only KSL2010*/pRNE formed motility zones upon RNase E induction with 0.1% arabinose. In contrast, KSL2010*/pRNE without arabinose induction remained nonmotile. Similarly, the KSL2010*/pBAD, KSL2010*/pDM, and Δ*fliC* strains all remained nonmotile regardless of 0.1% arabinose induction (Fig. 1E). These data indicate that both RNase E itself and its catalytic activity are indispensable for FliC expression, flagellar filament formation, and bacterial motility.

Notably, the *rne*-null strain KSL2010*/pBAD, which overexpresses RNase G, remained nonflagellated and nonmotile (Fig. 1C–E), indicating that RNase G overexpression cannot substitute for RNase E in flagellation and motility in the *rne*-null strain. This was further confirmed by deleting *rng* in an *E. coli* K-12 MG1655 (CGSC#6300) variant (MG1655_mv; see below and SI Materials and Methods for strain construction), which resulted in similar motility levels to its isogenic wild type (Fig. 1F). This finding is consistent with a previous study on *Pseudomonas putida*, showing that *rng* deletion had no significant impact on bacterial motility (27).

### Wild-type RNase E fully restores flagellar biogenesis and motility of *rne* mutant strains under both aerobic and anaerobic conditions

**Fig. 2.**
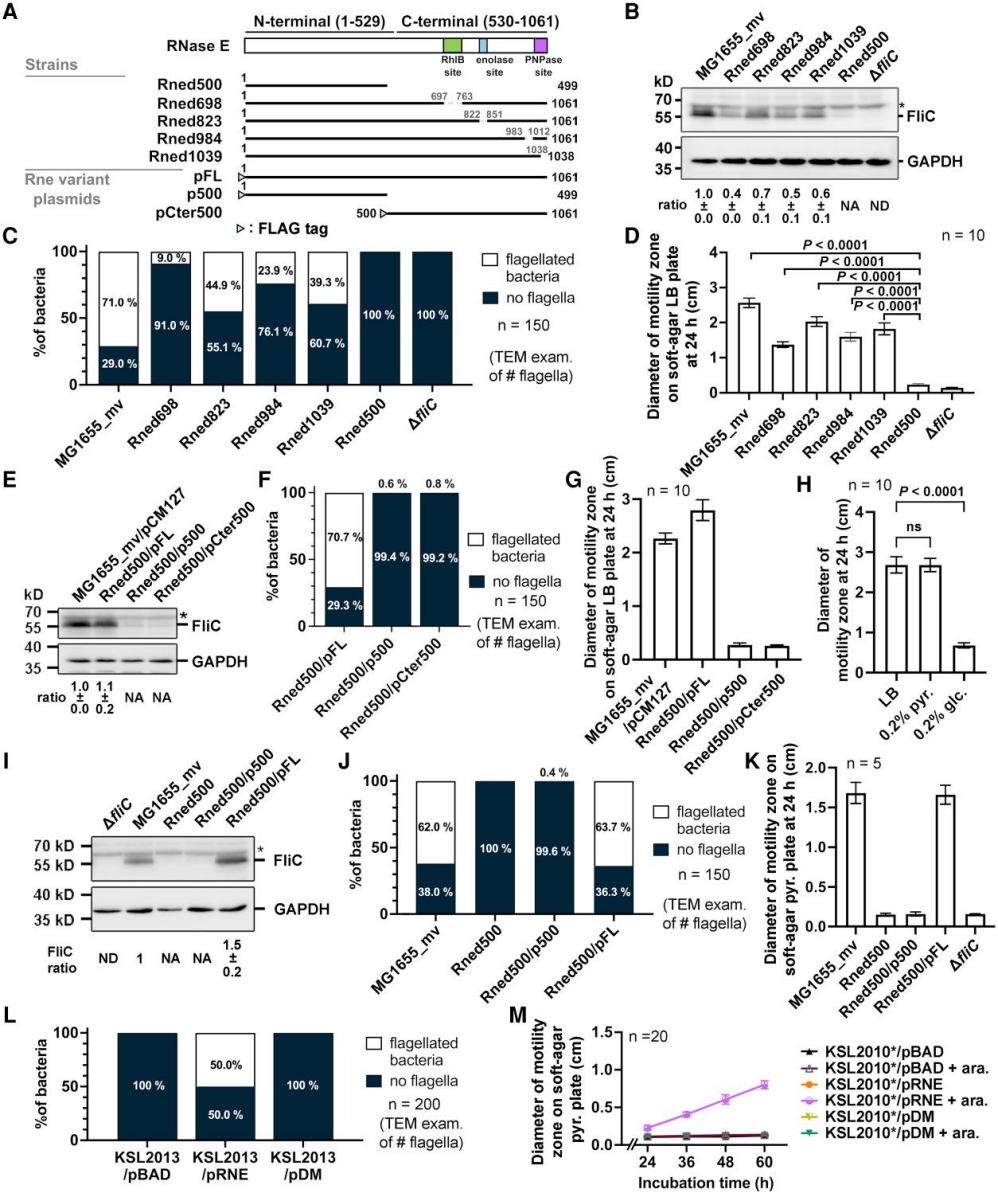
Disruption of RNase E C-terminal integrity or silencing its catalytic activity impairs flagella formation and motility in *E. coli* under aerobic and anaerobic conditions, both restored by wild-type RNase E complementation. A) Schematic representation of *rne* variants encoded by the chromosomes of *E. coli* strains and plasmids. Full-length RNase E with its microdomains is depicted above the schematics. Green rectangle, RhlB binding domain; blue rectangle, enolase binding domain; purple rectangle, PNPase binding domain; blue triangle, FLAG tag. The encoded amino acids and deleted binding sites are indicated by numbers. B, E, I) Western blot of FliC and GAPDH (loading control) in *rne* mutant strains (B, aerobic, LB) and RNase E complemented strains (E, aerobic, LB; I, anaerobic, LB with 0.2% pyruvate). MG1655_mvΔ*fliC* served as negative control. The pCM127, an empty vector plasmid, was used as a negative control for the pFL, p500, and pCter500 plasmids. Antibody against FliC, and GAPDH were used. Asterisk, the nonspecific signal of FliC antibody. Protein markers are indicated in kD. Relative FliC abundances are shown. Values are mean ± SD (*n* = 3 replicates). NA, not available; ND, not detected. Aerobic (+) and anaerobic (−) conditions are indicated. C, F, J, L) Quantification of flagellated/nonflagellated cells from TEM images of *rne* mutant strains (C, aerobic, LB), RNase E complemented strains (F, aerobic, LB; J, anaerobic, LB with 0.2% pyruvate), and KSL2010* variant strains (L, anaerobic, LB + 0.2% pyruvate and 0.1% arabinose). *n* = 150 or 200 cells/strain. D, G, H, K, M) Motility zone diameters of *rne* mutant and MG1655_mvΔ*fliC* strains (D, aerobic, LB), RNase E–complemented strains (G, aerobic, LB; K, anaerobic, LB with 0.2% pyruvate), and MG1655_mv with different carbon sources (H, aerobic, LB, LB with 0.2% pyruvate, LB with 0.2% glucose) after 24 h of incubation; and KSL2010* variant strains (M, anaerobic, LB + 0.2% pyruvate and with or without 0.1% arabinose (ara.)) for 60 hours of incubation (*n* = 5, 10 or 20 biological replicates as indicated; one-way ANOVA (*P* < 0.001), followed by Dunnett's post hoc test against the control group). Values are mean ± SD.

Full-length RNase E forms the RNA degradosome complex through its C-terminal domain (8, 9). To determine the impact of RNase E integrity on flagellar biogenesis and motility, we generated chromosomal *rne* mutants lacking coding regions for RNase E microdomains responsible for degradosome component binding (Fig. 2A).

*Escherichia coli* K-12 MG1655 stocks from the Coli Genetic Stock Center exhibit variable motility due to sequence modifications (e.g. insertion sequence [IS] elements) in the upstream region of the class I *flhDC* promoter (28). To study flagellar biogenesis and motility without the interference of IS elements (700–2,500 bp) (29), we modified the *flhDC* promoter region in MG1655 (CGSC#6300) by introducing a 69-bp sequence, creating MG1655_mv (mv, for motility variant), which exhibited a 6-fold increase in motility while remaining IS element-free (see SI Materials and Methods for details; Fig. S1). Since MG1655_mv was derived from the well-characterized CGSC#6300 background (30), and showed optimal motility, MG1655_mv was used as the parental strain for subsequent mutant generation. Additionally, the N3433-derived *rne*-null KSL2010* strain carries other mutations, including *relA* and *spoT* deletion (31), which repress flagellation and motility (32, 33). Therefore, KSL2010* is not ideal for studying flagellar biogenesis and function. The *rne* mutants derived from MG1655_mv included Rned500 (truncated after residue 499, removing part of the N-terminal domain residues 500–529 and the entire C-terminal domain residues 530–1,061), Rned698 (lacking the RhlB binding site), Rned823 (lacking the enolase binding site), Rned1039 (lacking the PNPase binding site), and Rned984 (lacking a control stretch of the C-terminal region) (see SI Materials and Methods for details). Western blotting showed a 40–70% reduction in FliC expression in Rned698, Rned823, Rned1039, and Rned984 compared with MG1655_mv, while Rned500 and the nonmotile negative control MG1655_mvΔ*fliC* showed no FliC expression (Fig. 2B). TEM analysis revealed that 71% of MG1655_mv cells were flagellated, whereas 9–44.9% of Rned698, Rned823, Rned984, and Rned1039 cells were flagellated. In contrast, Rned500 and MG1655_mvΔ*fliC* cells were nonflagellated (Fig. 2C). Motility assays further revealed that MG1655_mv formed a motility zone of 2.6 ± 0.1 cm. The Rned698, Rned823, Rned1039, and Rned984 strains formed smaller motility zones, ranging from 1.4 to 2.0 cm. In contrast, neither Rned500 nor MG1655_mvΔ*fliC* formed motility zones (Fig. 2D). We confirmed this finding in MG1655 variants containing IS elements at their *flhDC* promoter. Motility assay and TEM analysis demonstrated that Rned500 severely impairs flagellation and motility in these variants compared with their isogenic wild-type strains (Fig. S2). These results indicate that wild-type RNase E is required for normal flagellar biogenesis and bacterial motility, regardless of sequence modifications in the upstream region of the class I *flhDC* promoter.

To confirm the requirement of wild-type RNase E, we complemented Rned500 with low-copy-number plasmids, pFL (expressing wild-type RNase E, residues 1–1,061 aa), p500 (expressing C-terminal-truncated RNase E, residues 1–499 aa) or pCter500 (expressing N-terminal-truncated RNase E, residues 500–1,061 aa; Fig. 2A), respectively, generating the Rned500/pFL, Rned500/p500, and Rned500/pCter500 strains. Expression of these RNase E variants was driven by the *rne* native autoregulating promoter (34, 35). Western blot, TEM analysis, and motility assay showed restoration of FliC expression, flagella formation, and motility in Rned500/FL but not Rned500/p500 and Rned500/pCter500 strain (Fig. 2E–G). Taken together, these results demonstrate that wild-type RNase E is required for *E. coli* to maintain normal FliC expression, flagella formation, and bacterial motility. Expression of the N-terminal and C-terminal domains in *trans* is insufficient, indicating that both regions must function in *cis* as part of a single polypeptide. Consequently, mutations that disrupt RNase E integrity result in reduced FliC expression, flagella formation, and motility. Furthermore, deletion of the entire C-terminal domain of RNase E eliminates flagellar biogenesis and motility. Based on these findings, subsequent experiments investigating RNase E–dependent regulation of flagellar biogenesis and motility focused on the wild-type strain, Rned500, Rned500/pFL, and Rned500/p500. The Rned500/pCter500 strain was excluded from further analysis due to its lack of functional complementation.

Given that *E. coli* commonly grows in the intestines of warm-blooded organisms, which are typically low-oxygen environments (36), we examined whether RNase E is required for flagellar biogenesis and motility under anaerobic conditions, as it is under normal aerobic conditions. Under anaerobic growth, *E. coli* cells convert most of the carbon source (e.g. glucose and pyruvate) to several organic acids and biomass. However, due to the lack of oxygen as an electron acceptor during metabolism, glucose is partially oxidized, with acetate being excreted as a major reduced end product (37). Given that LB broth lacks fermentable sugars for anaerobic growth and that glucose inhibits flagellar biogenesis (38, 39), we assessed whether pyruvate could serve as an alternative carbon source under anaerobic conditions without affecting flagellar biogenesis. Motility assays under normal aerobic conditions showed that MG1655_mv motility was significantly inhibited in LB plus 0.2% glucose, as expected (38), but remained similar between LB plus 0.2% pyruvate and pure LB (Fig. 2H), indicating that pyruvate did not repress motility under normal aerobic conditions. Therefore, we used pyruvate as a carbon source for subsequent anaerobic experiments.

Using MG1655_mv, Rned500, Rned500/p500, and Rned500/pFL strains, all grown anaerobically in LB plus 0.2% pyruvate, subsequent western blotting, TEM analysis, and motility assay showed that both MG1655_mv and Rned500/pFL exhibited FliC expression, flagella formation, and motility under anaerobic conditions, whereas Rned500, Rned500/p500, and Δ*fliC* strains did not (Fig. 2I–K). The requirement of wild-type RNase E was confirmed in KSL2010* strain, showing that only the strain complemented with wild-type RNase E but not catalytic-null mutant could restore flagellar formation and motility (Fig. 2L and M). Together, these results indicate that RNase E and its catalytic activity are essential for flagellar biogenesis and bacterial motility under both aerobic and anaerobic growth conditions.

### *Escherichia coli* expressing wild-type RNase E, but not C-terminal truncated RNase E, triggers TLR5-NF-κB innate immune response during *E. coli*–human cell interactions

**Fig. 3.**
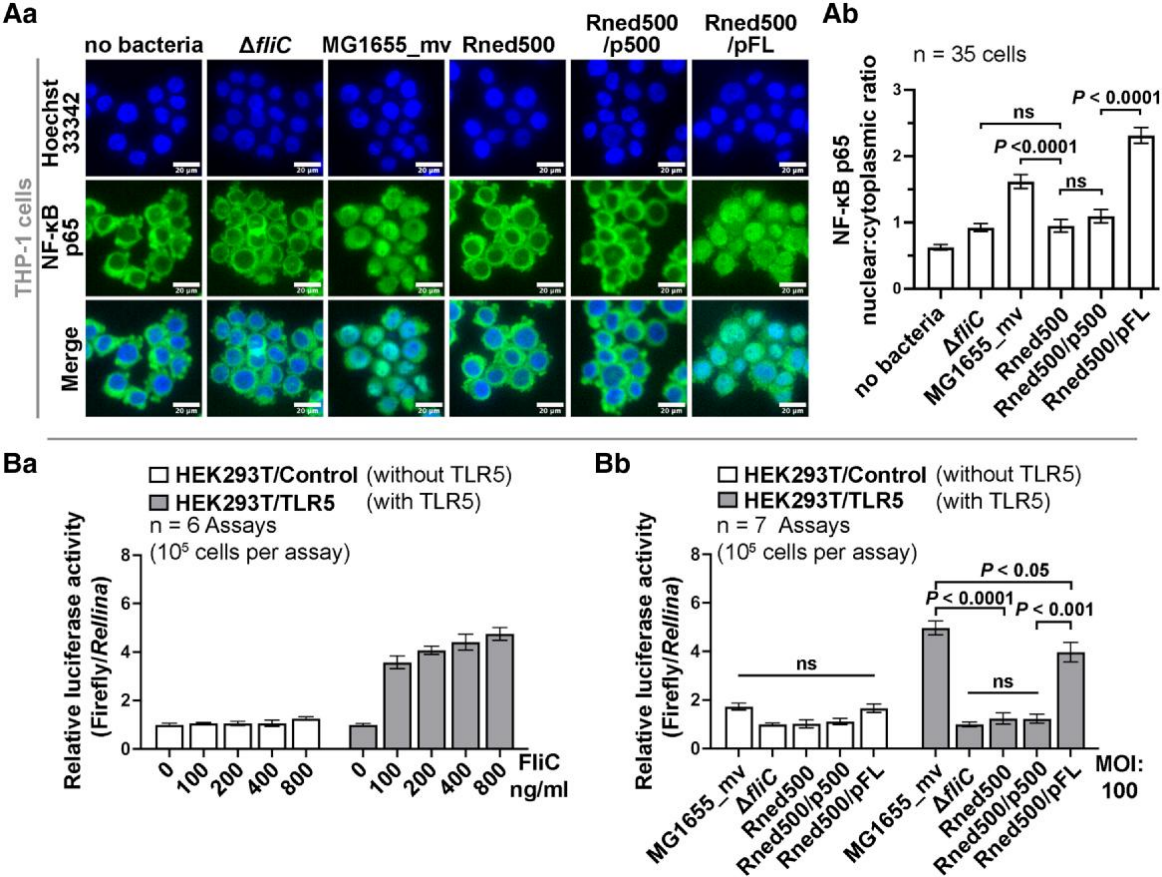
RNase E–dependent flagellar expression correlates with TLR5-mediated NF-κB activation in *E. coli*–challenged THP-1 and HEK293T cells. Aa) High content fluorescence microscopy of NF-κB (green) and Hoechst-stained nuclei (blue) in THP-1 cells challenged by heat-killed MG1655_mv, Rned500, Rned500/p500, Rned500/pFL, and MG1655_mvΔ*fliC* (negative control) *E. coli* strains. Scale bars, 20 μm. Ab) Quantification of the NF-κB nuclear:cytoplasmic ratio from NF-κB immunostaining microscopy images (*n* = 35 individual THP-1 cells; one-way ANOVA, *P* < 0.001, followed by Tukey's honestly significant difference (HSD) post hoc test). Ba, Bb) NF-κB activity of HEK293T/Control (white bar) and HEK293T/TLR5 (gray bar) cells stimulated with purified flagellin (0 to 800 ng) (Ba) or *E. coli* strains (MOI = 100) (Bb) for 6 h (*n* = 6–7 biological repeats; each repeat with three technical replicates; two-way ANOVA followed by Tukey's HSD post hoc test). Values are mean ± SD.

Our results showed that MG1655_mv and Rned500/pFL (complemented with wild-type RNase E), but neither Rned500 nor Rned500/p500 (complemented with C-terminal-truncated RNase E), formed flagella (Fig. 2). Bacterial flagella, through their flagellin (FliC) component, are known to activate host innate immune responses via the TLR5-NF-κB signaling pathway (23, 24). Activated NF-κB translocates into the nucleus and induces proinflammatory gene expression (40). To determine whether RNase E–dependent flagellar expression influences the host cell TLR5-dependent NF-κB signaling pathway during bacterial–host cell interactions, we first analyzed the cellular distribution of NF-κB in THP-1 cells (a human monocyte cell line) challenged by heat-killed MG1655_mv, Rned500, Rned500/p500, Rned500/pFL, or MG1655_mvΔ*fliC* (negative control) strains. Immunofluorescence imaging revealed that, without bacterial challenge, NF-κB was primarily cytoplasmic in THP-1 cells, with a nuclear:cytoplasmic ratio of 0.6 ± 0.0 (Fig. 3Aa and Ab). Upon challenge with MG1655_mv or Rned500/pFL, NF-κB translocated into the nucleus, increasing the nuclear:cytoplasmic ratio to 1.6 ± 0.1 and 2.3 ± 0.1, respectively. In contrast, cells challenged with Rned500, Rned500/p500, or MG1655_mvΔ*fliC* showed only minimal NF-κB nuclear translocation, with nuclear:cytoplasmic ratios of 1.0 ± 0.1, 1.1 ± 0.1, and 0.9 ± 0.1, respectively (Fig. 3Aa and Ab). These results indicate that RNase E–dependent flagellar expression contributes to NF-κB activation during *E. coli*–host cell interactions.

To determine whether this activation of NF-κB is mediated by TLR5, we performed a TLR5-NF-κB dual-luciferase reporter assay in transfected HEK293T cells, a human embryonic kidney cell line (see SI Materials and Methods for details). Firefly luciferase activity served as a reporter of TLR5-NF-κB signaling transduction, while *Renilla* luciferase activity measured plasmid transfection efficiency and ensured proper interpretation of normalized results (41). We used HEK293T cells because of their inherently poor response to flagellin stimulation and high plasmid transfection efficiency (42).

The dual-luciferase reporter assay showed that HEK293T/TLR5 cells (transfected with a TLR5-expressing plasmid) responded to purified flagellin stimulation (100 to 800 ng/mL), with relative luciferase activities ranging from 3.6 to 4.7 (Fig. 3Ba). In contrast, HEK293T/control cells (transfected with a control plasmid) showed no response upon flagellin stimulation (Fig. 3Ba). This result confirmed that flagellin-stimulated firefly luciferase induction in transfected HEK293T cells depended on TLR5 expression. To examine whether the observed NF-κB activation in THP-1 cells challenged by MG1655_mv and Rned500/pFL was mediated by TLR5 (Fig. 3Aa and Ab), we challenged the established transfected HEK293T/control and HEK293T/TLR5 cell system with the same *E. coli* strains as previously used. Dual-luciferase reporter assay revealed that HEK293T/TLR5 cells challenged by either MG1655_mv or Rned500/pFL, but not Rned500, Rned500/p500, or MG1655_mvΔ*fliC* strains, exhibited strong firefly luciferase induction (Fig. 3Bb). Additionally, HEK293T/control cells exhibited poor responses to all *E. coli* strains. These results indicate that NF-κB activation triggered by both MG1655_mv and Rned500/pFL cells relies on TLR5 expression in the HEK293T cells. Our findings demonstrate that wild-type RNase E–expressing *E. coli* (MG1655_mv and Rned500/pFL), which produce flagella, trigger TLR5-mediated NF-κB activation in human cells, whereas *E. coli* expressing C-terminal-truncated RNase E (Rned500 and Rned500/p500) do not. Together, results shown in Fig. 3 demonstrate that the ability of *E. coli* to elicit TLR5-mediated NF-κB signaling in human cells depends on RNase E–dependent flagellar expression.

### RNase E C-terminal truncation of Rned500 strain dysregulates class I and II flagellar gene expression by increasing mRNA stability

Our study demonstrated that RNase E, an essential endoribonuclease that influences RNA abundance and stability (10), is essential for flagellar biogenesis, which in turn enables flagellin-dependent activation of the TLR5-mediated NF-κB immune response (Figs. 1–3). These findings prompted us to further understand why RNase E C-terminal truncation impairs flagellar biogenesis, motility, and consequently affects the human cell TLR5-NF-κB response. *Escherichia coli* flagellar biogenesis requires ∼50 genes, arranged in 14 operons, whose expression is organized into a three-tiered transcriptional cascade (classes I–III; Fig. S3) (18). Class I genes encode the master regulator FlhDC, which activates class II gene expression. Class II genes encode proteins involved in the construction of the flagellar type III secretion system (T3SS; e.g. FlhA, FlhB), the hook-basal body (HBB; e.g. FliF, FliM, and FliI), the flagellar sigma factor FliA (required for class III gene expression), and the anti-sigma factor FlgM (which inhibits FliA). Class III genes encode the filament protein FliC, stator proteins, and chemotaxis proteins. To investigate the posttranscriptional regulation of RNase E on flagellar gene expression, we combined RNA sequencing (RNA-seq) and RNA half-life analyses to examine the abundance and stability of flagellar transcripts. Total RNA was isolated from *E. coli* MG1655_mv, Rned500, and Rned500/pFL strains grown separately on LB soft-agar plates under aerobic conditions. Subsequently, the total RNA was mixed with RNA spike-ins for normalization before RNA-seq analysis (see SI Materials and Methods for details) (43).

**Fig. 4.**
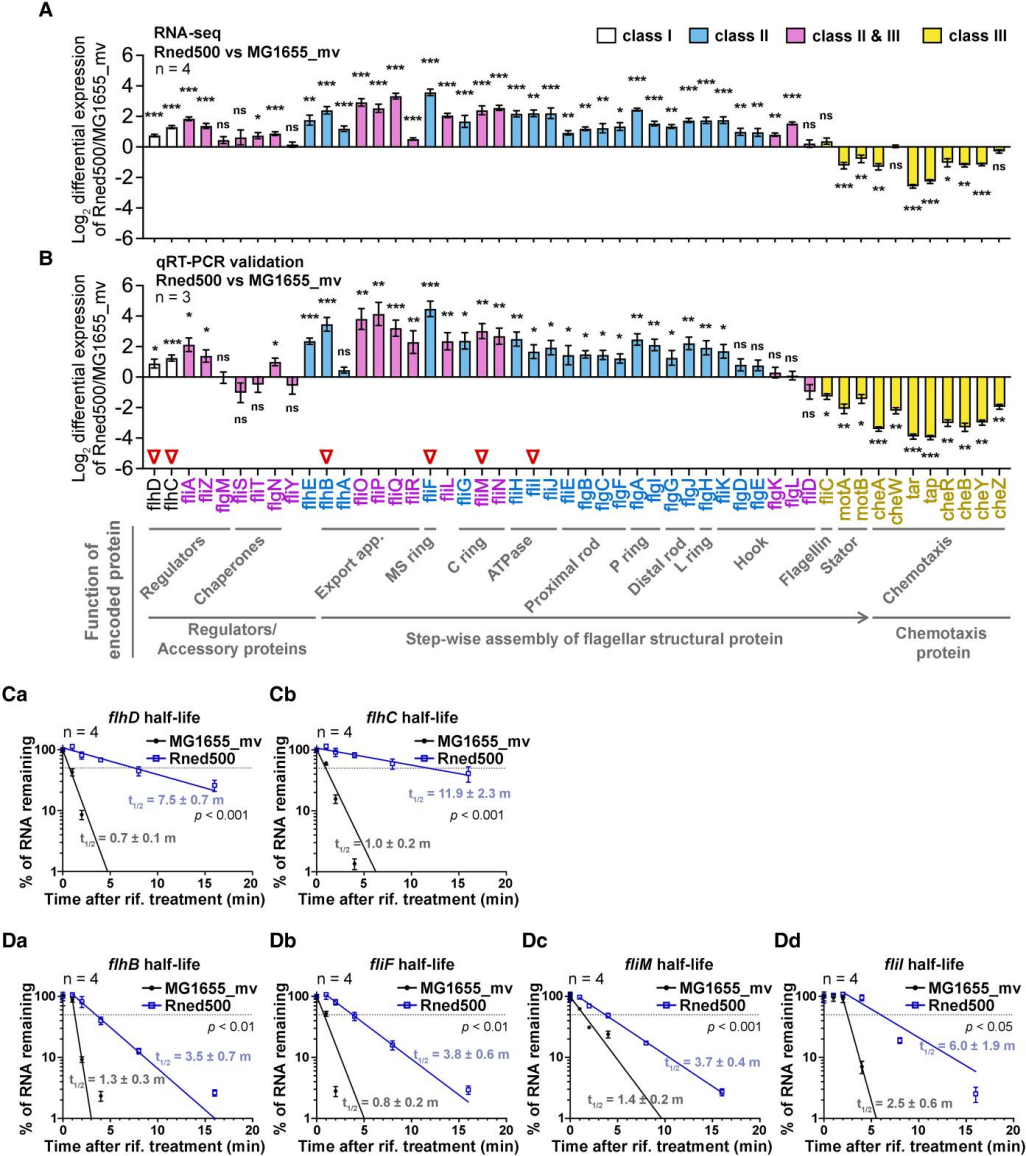
Dysregulation of flagellar gene expression and increased flagellar mRNA half-lives in the Rned500 mutant. A, B) Differential mRNA levels in Rned500 mutant compared with parental MG1655_mv, as determined by RNA-Seq analysis (A), and qRT-PCR validation (B). Genes are arranged according to the function of their encoded proteins, as described below the figure. Values are mean ± SD of log_2_ relative expression fold-change (*n* = 3 or 4 biological replicates as indicated; multiple unpaired *t-*test). ***P < 0.001; **P < 0.01; *P < 0.05; ns, *P* ≥ 0.05. White bar, class I genes; blue bar, class II genes; pink bar, class II&III genes; yellow bar, class III genes; red inverted triangles, genes selected for RNA half-life analysis and GFP translational fusion assays. Ca–Dd) Remaining RNA abundances of *flhD* (Ca), *flhC* (Cb), *flhB* (Da), *fliF* (Db), *fliM* (Dc), and *fliI* (Dd) transcripts in MG1655_mv and Rned500 plotted against time after rifampicin treatment. Dashed line, 50% remaining RNA. Half-lives (*t*_1/2_) are shown in minutes (m). Values are mean ± SD (*n* = 4 biological replicates; nonlinear regression fit; unpaired *t*-test).

RNA-seq analysis showed that mRNA levels for most of the class I and II genes were increased in the Rned500 relative to MG1655_mv, whereas they were decreased for the class III genes (Fig. 4A). We validated this finding with qRT-PCR of the MG1655_mv and Rned500 strains grown under normal aerobic conditions in LB liquid culture. Consistently, the majority of mRNAs of both classes I and II were up-regulated 0.6- to 23.1-fold (log_2_ −0.9 to 4.5), with the mRNA encoding the MS-ring structural protein FliF showing the highest upregulation. Conversely, class III mRNAs were downregulated, ranging from 0.1- to 0.4-fold (log_2_ −1.3 to −4.0), in Rned500 compared with MG1655_mv (Fig. 4B and Table S5). To determine if up-regulation of the class I and II flagellar mRNAs in Rned500 was attributable to changes in RNA stability, we determined the RNA half-lives of two class I genes (*flhD* and *flhC*, flagellar master regulators) and four class II genes (*flhB*, *fliF*, *fliM*, and *fliI*, structural proteins for initial HBB assembly) in MG1655_mv and Rned500 strains (marked by red triangles in Fig. 4B). qRT-PCR analysis revealed that the half-life of all these mRNAs increased by 2.4- to 11.9-fold in Rned500 relative to MG1655_mv (Fig. 4Ca–Dd). This indicates that the accumulation of class I and II transcripts in the Rned500 is due to increased RNA stability.

When wild-type RNase E was expressed in the Rned500 strain (Rned500/pFL), mRNA levels of class I, II, and III genes were restored to parental MG1655_mv levels (Fig. S4). These data demonstrate that RNase E C-terminal truncation leads to flagellar transcriptional cascade dysregulation, which can be fully restored by full-length RNase E. Collectively, these results indicate that RNase E regulates the abundance of class I and II flagellar mRNAs by modulating their RNA stability.

### Increased mRNA abundance and stability but decreased protein levels of the class I–encoded flagellar regulator and class II–encoded HBB structural proteins in Rned500

**Fig. 5.**
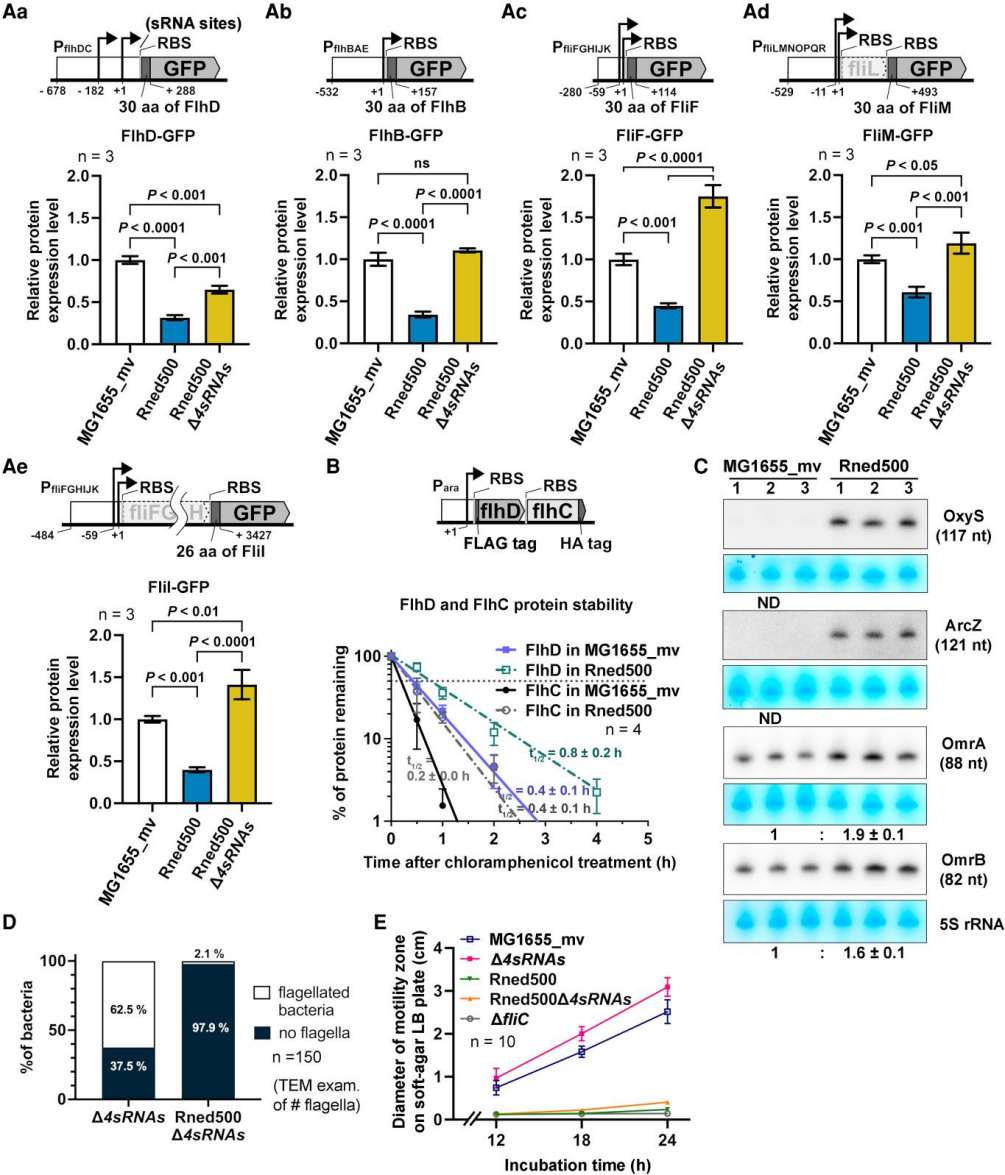
Deletion of negative regulatory sRNAs restores flagellar protein synthesis but fails to restore flagella formation and motility in Rned500. Aa-Ae) Relative protein abundances from GFP translational reporter assay on FlhD (Aa), FlhB (Ab), FliF (Ac), FliM (Ad), and FliI (Ae) in MG1655_mv, Rned500, and Rned500Δ*4sRNAs*. Values are mean ± SD (*n* = 4 biological repeats; unpaired *t*-test). Schematics above each graph show flagellar GFP fusion constructs, including the native promoter, RBS, and the coding region of first 26 or 30 amino acids of each protein. Numbers indicate the nucleotide regions included in the construct relative to the transcription start site (+1). sRNA regulatory sites on the *flhD* transcript are indicated. B) Percentage of remaining FLAG-FlhD and FlhC-HA protein levels in MG1655_mv and Rned500 were plotted against time after chloramphenicol treatment. Dashed line, 50% protein remaining. Half-lives (*t*_1/2_) are shown in hours (h). Values are mean ± SD (*n* = 4 replicates; nonlinear regression fit). C) Northern blot of OxyS, ArcZ, OmrA, and OmrB sRNAs in MG1655_mv and Rned500 during exponential growth. Methylene blue–stained 5S rRNA used as a loading control. Relative expression levels are shown. Values are mean ± SD (*n* = 3 biological replicates). n.d., nondetectable. D) Quantification of flagellated/nonflagellated cells from TEM images of sRNA-deletion strains (*n* = 150 cells/strain). E) Motility zone diameters of sRNA-deletion strains on LB soft-agar over 24 h incubation. Values are mean ± SD (*n* = 10 biological replicates). ND, not detected.

To investigate whether RNA accumulation increases protein expression, a GFP translational fusion assay was performed in MG1655_mv and Rned500 strains. Each strain carried an individual low-copy plasmid encoding a GFP fusion to the C-terminal end of the first 26 or 30 amino acids of either the class I protein FlhD or one of the class II proteins (FlhB, FliF, FliM, or FliI; Fig. 5Aa–Ae). These flagellar fusion constructs were driven by their native promoters, and the subsequent transcripts contained their own ribosome binding sites (RBS) and 5′ untranslated regions (5′ UTRs) regulatory elements, including small RNA (sRNA) regulatory sites. The resulting flagellar GFP fusion protein was used to measure the relative protein level. Western blot analysis, normalized against TolA (loading control; Fig. S5A), showed that the relative abundance of the class I FlhD-GFP fusion protein in Rned500 was reduced to 30% compared with MG1655_mv (Fig. 5Aa). This reduction was further corroborated by western blot analysis of native FlhD bearing an N-terminal FLAG tag and expressed from its native promoter, which also exhibited a reduced protein level in the Rned500 strain (Fig. S5B). This decreased protein level in Rned500 could result from either reduced protein synthesis or increased protein degradation (i.e. reduced protein stability/half-life).

Therefore, we further examined the stability of native class I FlhD and FlhC proteins. To bypass native transcriptional regulation, we constructed a low-copy-number plasmid encoding N-terminal FLAG-tagged FlhD and C-terminal HA-tagged FlhC under the control of an arabinose-inducible promoter. Protein half-life was assessed by treating cells with chloramphenicol to inhibit protein synthesis in the presence of arabinose. Western blot showed that FLAG-FlhD and FlhC-HA protein half-lives doubled (from 0.4 to 0.8 and 0.2 to 0.4 h, respectively) in Rned500 compared with MG1655_mv (Figs. 5B and S5G), indicating reduced protein degradation. These results rule out increased protein degradation as the cause of the reduced class I FlhD-GFP level observed in Rned500 and suggest that the decrease likely results from reduced protein synthesis.

Similarly, western blot of class II flagellar structural protein GFP fusions, normalized against TolA (loading control; (Fig. S5C–F), showed that the relative abundance in Rned500 was reduced to ∼30 to 60% compared with MG1655_mv (Fig. 5Ab–Ae), despite a 3.3- to 23.1-fold increase in mRNA abundance (Fig. 4A and B). Overall, these results demonstrate that despite increased abundance and stability of class I and II flagellar mRNAs, their corresponding GFP fusion protein abundance is greatly reduced in Rned500, correlating with its nonflagellated and nonmotile phenotype (Fig. 2).

### Restoration of flagellar protein abundance via negative regulatory sRNA deletion or plasmid-based flagellar protein overexpression in Rned500 fails to rescue flagella formation and motility

To determine whether the observed reduction in flagellar protein abundance in the Rned500 resulted from decreased protein synthesis via posttranscriptional repression, we examined the levels of known flagellar-associated sRNAs (44, 45), which posttranscriptionally regulate protein translation (46). Studies have shown flagellar-associated sRNAs primarily target the class I *flhDC* transcript. Specifically, McaS sRNA positively regulates its translation (45), while OxyS, ArcZ, OmrA, and OmrB sRNAs negatively regulate it (44). Northern blot analysis revealed increased accumulation of the negative regulatory sRNAs (OxyS, ArcZ, OmrA, and OmrB) in Rned500 compared with MG1655_mv (Fig. 5C). Conversely, McaS sRNA expression was undetectable in both MG1655_mv and Rned500 strains (data not shown), consistent with a previous report that McaS expression is low during exponential growth in MG1655 strain (45). These results exclude the possibility that McaS contributes to reduced flagellar protein levels and suggest that the decrease in protein levels in Rned500 is due to the accumulation of negative regulatory sRNAs. We deleted these negative regulatory sRNAs from Rned500 strain (Rned500Δ*4sRNAs*) and found that flagellar class I master regulator and class II structural protein GFP fusions were restored to MG1655_mv levels (Fig. 5Aa–Ae). These findings demonstrate that accumulation of negative regulatory sRNAs in the Rned500 strain posttranscriptionally represses flagellar class I and II GFP fusion protein expression.

Despite restoring the abundance of flagellar class I master regulator and class II structural protein GFP fusions in Rned500Δ*4sRNAs* to parental MG1655_mv levels, TEM analysis and motility assays showed that Rned500Δ*4sRNAs* remained nonflagellated and nonmotile, similar to Rned500 (Fig. 5D and E). This finding indicates that simply restoring the abundance of class I and II flagellar proteins is insufficient to rescue flagellar biogenesis and motility in the Rned500 background.

Similarly, plasmid-based overexpression of class I– and class II–encoded regulators in Rned500 failed to restore flagellar biogenesis and motility, despite an increase in FliC expression (Fig. S6). Taken together, these results demonstrate that while the accumulation of negative regulatory sRNAs contributes to reduced flagellar protein expression in the Rned500 strain, restoring class I and II flagellar protein levels, including master regulators and structural proteins crucial for initial HBB assembly, as well as FliC (Figs. 5 and S6), does not restore flagellar biogenesis and motility. This result suggests that either the flagellar protein assembly process or the protein exportation facilitated by flagellar chaperones is defective in Rned500.

### Impaired early flagellar assembly in Rned500 showing HBB structural protein FliF mislocalization and FlgM secretion defect

**Fig. 6.**
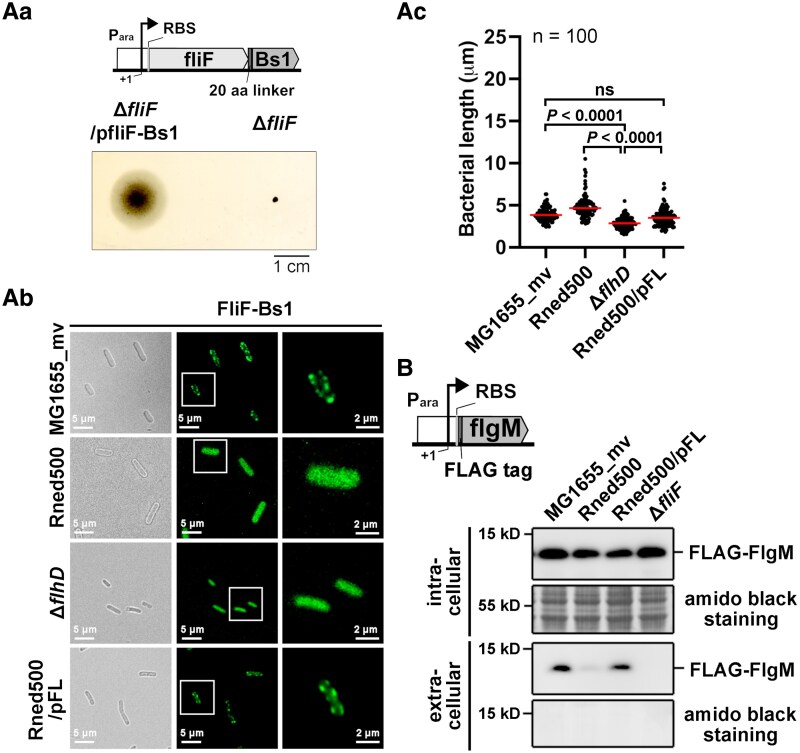
Defects in hook-basal body assembly and FlgM secretion in Rned500. Aa) Representative image of the motility zone of MG1655_mvΔ*fliF* and MG1655_mvΔ*fliF*/fliF-Bs1 on an LB soft-agar plate supplemented with 0.2% arabinose for FliF-Bs1 induction after 36 h of incubation. Scale bar, 1 cm. Ab) Representative fluorescence microscopy images and respective brightfield images of FliF-Bs1 in MG1655_mv, Rned500, Rned500/pFL, and MG1655_mvΔ*flhD* (negative control) under 0.2% arabinose induction (*n* = 3 biological replicates). Scale bars, 5 and 2 μm, as indicated. Areas within white boxes are enlarged at right. Ac) Dot plot of bacterium length (μm) for strains used in (Ab) (*n* = 100 cells/strain; one-way ANOVA). Red line, median. B) Western blot of intracellular and extracellular (secreted) FLAG-FlgM of MG1655_mv, Rned500, and Rned500/pFL. MG1655_mvΔ*fliF* used as negative control for FLAG-FlgM secretion (*n* = 3 replicates). GAPDH used as loading control. Antibody against FLAG tag and GAPDH were used. A schematic diagram depicting the FLAG-FlgM constructs is shown.

During stepwise assembly of flagella, MS ring formation and flagellar T3SS construction represent the earliest steps (47, 48). The MS ring, composed of FliF and formed on the bacterial inner membrane, serves as the foundation for further flagellar assembly and function (48). The export of flagellar proteins (FlgK/FlgL, FlgM, FliC, and FliD) for further flagellar filament construction requires the assistance of flagellar chaperones (FlgN, FliA, FliS, and FliT, respectively) (49–51). Given that class I *flhC* deletion leads to mislocalization of FliF (47), and that class I protein expression is reduced in Rned500 (Fig. 5Aa), we examined whether the FliF was properly localized to the membrane for assembly in this strain. We used fluorescence microscopy to analyze the localization of full-length FliF fused at its C-terminus to the Bs1 fluorescent protein (FliF-Bs1), which retained all native FliF transmembrane domains. This fusion protein was encoded on a low-copy-number plasmid (pfliF-Bs1 plasmid) under the control of an arabinose-inducible promoter. The functionality of the FliF-Bs1 fusion protein was validated by its ability to restore motility in MG1655_mvΔ*fliF* (Fig. 6Aa). Subsequently, we examined the cellular localization of FliF-Bs1 in MG1655_mv, Rned500, Rned500/pFL, and nonflagellated MG1655_mvΔ*flhD* (negative control, lacking class II–III protein expression due to class I deletion) strains. Fluorescence microscopy revealed membrane-localized FliF-Bs1 puncta in MG1655_mv but a diffuse distribution in Rned500 and MG1655_mvΔ*flhD* (Fig. 6Ab). The diffuse distribution of FliF in class I *flhD* deletion control strain is consistent with a previous report in a class I *flhC* deletion control strain (47). Complementation of Rned500 with wild-type RNase E (Rned500/pFL) restored membrane-localized FliF-Bs1 puncta. These findings indicate that the Rned500 strain lacks proper FliF membrane localization, suggesting a defect in early flagellar HBB assembly. (Notably, MG1655_mvΔ*flhD* cells were significantly smaller, consistent with a previous report linking *flhD* deletion to reduced cell size [Fig. 6Ac] (52).)

A mature flagellar HBB is required for both FlgM secretion and construction of the flagellar axial filament structure (53–55). The observed mislocalization of FliF in Rned500 strain suggested a defect in early flagellar HBB assembly (Fig. 6Ab), potentially resulting in the absence of FlgM secretion. To examine FlgM secretion, we introduced a low-copy-number plasmid encoding N-terminal FLAG-tagged FlgM under control of an arabinose-inducible promoter (pFlgM plasmid) in MG1655_mv, Rned500, Rned500/pFL and MG1655_mvΔ*fliF* (negative control, lacking HBB). FlgM secretion assay (see SI Materials and Methods for details) showed extracellular FLAG-FlgM only in MG1655_mv and Rned500/pFL, but not in Rned500 or MG1655_mvΔ*fliF*, despite the similar intracellular FLAG-FlgM levels among these strains (Fig. 6B). Collectively, the absence of membrane-localized FliF, coupled with the lack of FlgM secretion in the RNase E C-terminal-truncated Rned500 strain, indicates a defect in mature flagellar HBB formation, specifically during early assembly steps. This suggests that wild-type RNase E is required for proper HBB assembly. Consequently, the HBB assembly defect in the Rned500 strain explains its nonflagellated, nonmotile phenotype and its failure to trigger the TLR5-NF-κB response during *E. coli*–host cell interactions (Figs. 1–3).

## Discussion

This study discovers that C-terminal truncation of RNase E leads to flagellar mRNA/sRNA accumulation but reduces protein levels via sRNA-mediated repression. This truncation also disrupts FliF membrane localization, leading to defective early HBB assembly, which consequently impairs flagellar protein export, filament formation, and overall flagellar biogenesis, resulting in drastically reduced motility and the ability to trigger human cells TLR5-NF-κB immune response during *E. coli*–host cell interactions. The defects in early HBB assembly, flagellar biogenesis, motility, and inability to trigger human cells TLR5-NF-κB activation observed in the Rned500 strain were rescued only by wild-type RNase E expression, not by ectopic expression of flagellar proteins. Together, our findings demonstrate the crucial role of RNase E in *E. coli* flagellar stepwise assembly during its biogenesis and function. This highlights the importance of posttranscriptional regulation within the flagellar transcriptional cascade, where RNase E, through its catalytic activity, fine-tunes the levels of flagellar class I to III mRNAs and flagellar-associated sRNAs, which maintains proper flagellar protein homeostasis for its assembly.

### Posttranscriptional regulation of the flagellar cascade in Rned500

Our result showed that class I mRNAs accumulated in Rned500 due to increased transcript stability (Fig. 4Ca and Cb), but this did not increase corresponding protein levels (Figs. 5Aa and S5B). The repression of class I protein expression in Rned500 can be attributed to the accumulation of four negative regulatory sRNAs (OxyS, ArcZ, OmrA, and OmrB), rather than reduced protein degradation (Fig. 5B and C). Although deletion of these sRNAs in Rned500 (Rned500Δ*4sRNAs*) restored class I protein levels (Figs. 5Aa and S5B), cells remained nonflagellated and nonmotile (Fig. 5D and E). Similarly, ectopic class I protein expression in Rned500 failed to restore flagellation and motility (Fig. S6Aa–Ac).

Given that class I proteins activate the transcription of class II genes, a reduction in class I protein abundance would be expected to result in decreased levels of class II mRNAs. Nonetheless, 27 out of 37 class II mRNAs accumulated in Rned500 strain (Fig. 4A and B). RNA stability analysis of four of these mRNAs revealed a 2.2- to 4.8-fold longer half-life (Fig. 4Da–Dd), indicating that their accumulation results from increased RNA stability, not transcriptional regulation by class I proteins. Although an increase in class II mRNAs would be expected to result in increased class II protein abundance, GFP fusions of four class II genes (*flhB*, *fliF*, *fliM*, *fliI*), as well as endogenous FliA protein detected by an anti-FliA antibody (Fig. S6Aa, lanes 1 and 4), showed reduced protein synthesis in Rned500 (Fig. 5Ab–Ae). Previous studies identified certain sRNAs that limit specific class II gene expression (56–58). We did not further delete these class II–associated sRNAs because class II GFP fusion protein levels in the Rned500Δ*4sRNAs* strain were already restored to the parental MG1655_mv levels, yet the cells remained nonflagellated and nonmotile (Fig. 5Ab–Ae). Similarly, plasmid-based overexpression of class II protein in Rned500 also failed to restore flagellation and motility (Fig. S6Ba–Bc). Together, increased class I or class II protein levels in Rned500 are insufficient for flagellation and motility.

A possible explanation for increased mRNA but reduced protein levels of class I and II genes in Rned500 is decreased translation efficiency. A recent study using an *rne* temperature-sensitive strain (Δ*rne*-3071) demonstrated that RNase E cleaves the 5′ UTR region of nonflagellar transcripts (*eno*, *bcp*, and *srlD*), thereby exposing the ribosome binding site and enhancing translation efficiency (59). Moreover, a study in *Salmonella* identified RNase E cleavage sites in the 5′ UTR region of several flagellar transcripts, including *flhD*, *flgM*, *flgA*, *flgK*, *fliY*, *fliB*, *fliD*, *fliF*, and *fliL* (as reported in their supplemental information) (60). Although previous reports showing RNase E cleavage in the 5′ UTR region of *flhD* and *fliD* reduced the FlhD-LacZ and FliD-CAT fusion protein level, respectively (19, 21), further investigation is needed to determine whether an RNase E mutant with reduced activity affects the cleavage in 5′ UTR region and, consequently, reduces the translation efficiency and expression of some of these flagellar transcripts.

In contrast to class I and II mRNAs, we observed a significant down-regulation of class III mRNAs, including *fliC* transcript, in Rned500 (Fig. 4A and B). This decreased mRNA expression may result from reduced transcription, potentially caused by lower class II protein levels or the control of the FliA/FlgM molecular checkpoint, which coordinates class III gene expression with completion of HBB assembly (53, 54). The observed defect in HBB assembly, caused by FliF mislocalization and the subsequent failure of FlgM secretion in the Rned500 strain, results in the inhibition of FliA activity and prevents the expression of class III genes (Figs. 4 and 6). Given that FliF mislocalizes in class I *flhD* or *flhC* deletion strains (Fig. 6) (47) and class I protein expression is reduced in Rned500 (Fig. 5Aa), the FliF mislocalization is likely an indirect effect of reduced class I protein levels within this strain.

### Regulation of RNase E catalytic activity and its role in flagellar biogenesis

It is known that the activity of RNase E is regulated by autoregulation of its own expression (34, 35), membrane attachment in response to growth conditions (12, 61, 62), or interactions with inhibitory proteins (63–65). One of the reasons why RNase E activity needs to be regulated is that its adventitious overexpression or increased enzymatic activity causes cellular toxicity, leading to growth retardation (66, 67). However, the full scope of why RNase E activity requires tight regulation remains unclear. In *Salmonella*, deleting the RNase E inhibitory protein gene *rraA* resulted in decreased bacterial motility, invasion ability, and cytotoxicity in human cervical epithelial carcinoma cell infections (21), implicating RNase E activity in virulence-associated behaviors. Furthermore, *E. coli* RNase E was shown to detach from the cellular membrane under anaerobic conditions (12)—a common environment encountered during host infection. Since membrane association enhances RNase E catalytic activity (61, 62), this detachment under anaerobiosis thus reduces its activity, leading to stabilization of the sRNA DicF (12), a key virulence factor required for host colonization (68).

Environmental signals encountered during host infection are also known to suppress flagellar expression. For instance, bacteria often turn off flagella expression after penetrating host protective barriers (e.g. mucus layers) to reduce immune detection via flagellin sensing (69, 70). Concurrently, the host releases nitric oxide (71), which alters the redox potential of the gut environment and promotes shifts in bacterial metabolism toward anaerobic respiration (72). These conditions may reduce RNase E membrane association and thus catalytic activity.

In this study, we show that RNase E catalytic activity is essential for flagellar biogenesis and motility under both aerobic and anaerobic conditions (Figs. 1 and 2L and M). While we do not directly test environmental modulation of RNase E during infection, our findings, together with prior studies, suggest that RNase E activity may serve as a point of integration between environmental cues and flagellar regulation. This potential regulatory link warrants further investigation to clarify how RNase E contributes to bacterial adaptation and immune evasion during host colonization.

### RNase E and flagellar regulation in diverse bacteria

Our results demonstrated that RNase E C-terminal microdomain mutations did not completely abolish flagellar biogenesis and motility (Fig. 2), indicating these microdomains are not strictly required. In contrast, complete truncation of the C-terminal region (e.g. Rned500) abolishes flagellar biogenesis and motility (Fig. 2), as does the catalytic-null mutant (D303N/D346N) (Fig. 1), indicating that both catalytic activity and the C-terminal scaffold contribute to flagellar regulation. Notably, our complementation experiments reveal that the C-terminal region must act in *cis* with the catalytic domain to support flagellar gene expression and motility, as trans-supplied C-terminal fragments (Rned500/pCter500) fail to restore these functions (Fig. 2). This likely reflects structural or spatial constraints necessary for proper degradosome assembly or substrate coordination. Although the precise molecular basis of this *cis*-dependence remains to be elucidated, its functional consequence is clearly demonstrated by our data.

Interestingly, RNase E homologs in some γ-Proteobacteria, such as *Legionella pneumophila* (a flagellated human pathogen (73)), lack a canonical C-terminal region yet still support motility. These homologs retain conserved catalytic motifs and membrane-tethering elements (Fig. S7). This observation suggests that an RNase E homolog extending to approximately residue 600 (in *E. coli* numbering, encompassing segment A) may represent a minimal functional unit sufficient for regulating flagellar biogenesis and motility, at least in certain bacterial species. The distinct *Legionella* architecture thus offers an intriguing comparative model for exploring the structural requirements of RNase E–mediated flagellar regulation across bacteria.

In contrast, gram-positive bacteria lack RNase E homologs and instead possess RNase J and RNase Y as functional analogs (74). Both RNases are components of degradosome-like complexes and are involved in the processing and degradation of numerous RNA molecules in flagellated *Bacillus subtilis* (75–77). However, no reports indicate these RNases regulate flagellar biogenesis or motility. Further research is required to determine whether these RNases also play regulatory roles in flagellar biogenesis.

## Materials and methods

Bacterial strains, plasmids, and oligonucleotide primer sequences used in this study are listed in Tables S1–S4. Additional materials and procedures for genetic construction, growth conditions, biochemical assays, reporter assays, imaging, physiological analysis, and statistical analysis are provided in the SI Materials and Methods.

## Supplementary Material

pgaf269_Supplementary_Data

## Data Availability

All data are included in the manuscript and/or Supplementary material.
