# Supplementary material for: The RNA degradation enzyme RNase E is essential for early flagellar assembly in Escherichia coli
Source: PNAS Nexus. 2025 Aug 18;4(9):pgaf269. doi: 10.1093/pnasnexus/pgaf269 (PMC12399333; doi:10.1093/pnasnexus/pgaf269)
Supplement: pgaf269_Supplementary_Data [file pgaf269_supplementary_data.docx]

**Supporting Information for**

The RNA Degradation Enzyme RNase E Is Essential for Early Flagellar Assembly in *Escherichia coli*

Wei-Syuan Wang^1,2^, Yu-Hsiang Chen^1,4^, Gunn-Guang Liou^1,3,4^, Oleg N. Murashko^1^, Sue Lin-Chao^1,2,5,*^

^1^Institute of Molecular Biology, Academia Sinica, Taipei 11529, Taiwan

^2^Molecular and Cell Biology, Taiwan International Graduate Program, Academia Sinica and Graduate Institute of Life Science, National Defense Medical Center, Taipei 11490, Taiwan

^3^Present address: Office of Research and Development, College of Medicine, National Taiwan University, Taipei 106319, Taiwan

^4^These authors contributed equally to this work

^5^Lead contact

*Corresponding author

**Email:**  mbsue@gate.sinica.edu.tw

**This PDF file includes:**

Supporting text

Figures S1 to S7

Tables S1 to S5

SI References

SI Materials and Methods

**Bacterial Strains, Plasmids, and Growth Conditions**

***Bacterial Strains Construction***

Bacterial strains used in this study are listed in Table S1. *E. coli* K-12 strain MG1655 from Coli Genetic Stock Center (CGSC) has different stocks exhibiting varying levels of motility (1). These different levels of motility result from different modifications upstream of *flhDC* promoter (1). It is known that sequence insertions or nucleotide mutations upstream of the *flhDC* promoter enhance bacterial motility (1, 2). To generate a bacterial strain with enhanced motility for studying flagellar biogenesis and bacterial motility, we modified the region upstream of the *flhDC* promoter by inserting a 69-base pairs (bp) insertion sequence (5'-CTG GAG CTG CTT CGA AGT TCC TAT ACT TTC TAG AGA ATA GGA ACT TCG GAA TAG GAA CTA AGG AGG ATA-3'; *FRT* sequence is underlined) at 99 nt upstream from the *flhDC* transcription start site in the MG1655 (CGSC #6300) strain (Figure S1Ac). We used a modified Datsenko and Wanner one-step chromosomal fusion method (3). Briefly, 40-nt homologous sequences to the region upstream of the *flhDC* promoter were designed on the forward and reverse primers for polymerase chain reaction (PCR) amplification of the Km cassette. The PCR product of the Km cassette was electro-transformed into the MG1655 strain pre-transformed with pKD46 carrying λ Red recombinase under the regulation of the arabinose promoter to obtain the intermediate MG1655_Km strain. We further removed the Km cassette from the region upstream of the *flhDC* promoter by introducing the pCP20 plasmid carrying Flp recombinase, which performs Flp-*FRT* recombination. This final strain was named as MG1655_mv strain and was used as the parental strain in this study.

To study the impacts of RNase E integrity on flagellar biogenesis and bacterial motility, we constructed *rne* microdomain mutant strains. Rne mutants with deletions of microdomains responsible for recognizing various degradosome components were introduced into the chromosome of the *E. coli* MG1655_mv parental strain. The microdomains were defined according to a previous publication (4). The C-terminal truncation mutant strain (Rned500), enolase binding site deletion mutant strain (Rned823), and 28 aa C-terminal segment deletion control strain (Rned984) in which association of RNA degradosome components is retained were all obtained from a previous publication (5) and introduced into the MG1655_mv parental strain of this study by P1 transduction (6). The Rned500 construct, which expresses RNase E residues 1–499, was previously characterized and shown to retain catalytic activity while lacking degradosome assembly functions. Notably, this truncation removes not only the entire C-terminal domain (residues 530–1061), but also the final 30 residues of the N-terminal domain (residues 500–529), as per the design in the original study. In the present work, this inherited construct served as a validated tool for assessing the functional importance of full-length RNase E.

The RhlB binding site deletion mutant (Rned698) and the PNPase binding site mutant strain (Rned1039) were constructed using a modified Datsenko and Wanner one-step chromosomal fusion method as mentioned above (3), except that pBAD-Rned698-762-Km and pBAD-Rned1039-1011-Km plasmids were used to amplify the Rne-Km cassette with the forward (rne_fw) and reverse (rne_rev) primers (Table S2). Then, 200 ng of each purified PCR product was electro-transformed into MG1655_mv/pkD46 cells. Transformed bacteria were incubated in LB medium for 1 hour at 37 ℃. *rne* mutant colonies were selected based on kanamycin resistance (Km, 25 µg/mL) on LB agar and incubated overnight at 37°C. After the primary selection, individual colonies were screened for ampicillin sensitivity to confirm the loss of the helper plasmid pKD46. Mutations were confirmed by DNA sequencing by the IBMS DNA Sequencing Core, Academia Sinica. P1 transduction (as described previously (6)) back to the MG1655_mv strain was performed to clean up the genomic background.

To generate the flagellar-associated sRNA quadruple deletion strains (MG1655_mvΔ*4sRNAs* and Rned500Δ*4sRNAs*), we deleted these four sRNAs one by one using one-step chromosomal fusion as described above. To generate MG1655_mvΔ*rng*, MG1655_mvΔ*flhD*, MG1655_mvΔ*fliA*, MG1655_mvΔ*fliC*, MG1655_mvΔ*fliF*, and Δ*fliC* variants in CGSC#6300, CGSC#7740, and CGSC#8237, we used P1 transduction to transfer these gene mutations from bacterial strains in Keio Collection (7). All mutant strains have been sequence-validated using the sequencing primers (Table S2).

To derive a *rne* chromosomal deletion (*rne*-null) strain, we modified the original KSL2010 strain (8) by exchanging the arabinose-induced pBAD-RNG plasmid (pSC101 *ori*, Km^R^) into the auto-expressed pga-RNG plasmid (pSC101 *ori*, Km^R^) in which expression of RNase G was driven by a GAPDH promoter to obtain the final KSL2010* strain. The derivative KSL2010* strain was selected by kanamycin resistance without supplemented of arabinose.

***Plasmids Construction***

To generate the working plasmids, we used pBAD-EBFP2 (Addgene Plasmid #14891, pBR322 *ori*, Ap*^R^*, ~20 copies/cell), pCM128 (pSC101 *ori*, Ap^R^, ~5 copies/cell) (9), and pT25 (p15A *ori*, Cm^R^, ~20 copies/cell) (10) as plasmid backbones. The control plasmids pBAD and pCM127 were obtained by removing the EBFP2-encoding and *lac* promoter sequences between the *EcoRI* and *BamHI* sites from pBAD-EBFP2 and pCM128, respectively.

To study the essentiality and catalytic activity of RNase E in flagellar biogenesis and motility, we constructed arabinose-inducible RNase E variants in the pBAD plasmid. For the arabinose-inducible pRNE plasmid, the *rne* gene was inserted between the *NdeI* and *EcoRI* sites. To obtain the catalytic-null RNase E-expressing plasmid pDM, D303N and D346N mutations were introduced into pBAD-RNE by site-directed mutagenesis (11). The D303N and D346N mutations were designed according to a previous crystal structure report (12).

To complement the Rned500 strain with wild-type, C-terminal-truncated RNase E or N-terminal truncated RNase E, we constructed pCM127 derivative plasmids, pFL (expressing wild-type RNase E), p500 (expressing the N-terminal 1-499 aa RNase E variant), and pCter500 (expressing the C-terminal 500-1061 aa RNase E variant). Expression of these RNase E variants was under the control of the native *rne* promoter for auto-regulation, preventing RNase E overproduction (13, 14). A FLAG-tag encoding sequence was fused to the 5'-end of these *rne* variant genes to derive N-terminal FLAG-tagged RNase E, allowing differentiation from chromosomally encoded RNase E in Western blot detection.

To study the effect of overexpressing flagellar class I and II proteins and FlgM secretion, we constructed arabinose-inducible pCM127 derivative plasmids: pflhDC (expressing FLAG-FlhD and FlhC-HA for protein distinction during detection), pfliA (expressing FLAG-FliA), and pFLAG-flgM (expressing N-terminal FLAG-tagged FlgM). Relevant genes were cloned between the *EcoRI* and *BamHI* sites. The functionality of plasmid-encoded FLAG-FlhD/FlhC-HA and FLAG-FliA was confirmed by FliC expression and motility restoration in MG1655_mvΔ*flhD* and MG1655_mvΔ*fliA*, respectively (FigureS6).

To determine the relative protein levels of flagellar genes in different strains, we constructed *gfp* fusion into pT25 to create translational fusion reporter plasmids (pflhD-GFP, pflhB-GFP, pfliF-GFP, pfliI-GFP, and pfliM-GFP). The sequence from the native promoter to the coding region of the first 26 or 30 amino acids (aa) of each gene was fused in-frame with the GFP-encoding sequence, replacing the T25 protein coding sequence upon insertion into the pT25 plasmid. GFP fusion reporter proteins were expressed under the control of their respective native promoters (*flhD*, *flhB*, *fliF*, *fliI*, and *fliM*). Additionally, we cloned the gene encoding native FlhD with an N-terminal FLAG tag, under the control of its native promoter, into the pCM127 plasmid (designated pPflhD) to validate the relative FlhD protein levels.

To study the cellular distribution of FliF, we constructed the coding sequence of the FliF-Bs1 fluorescent fusion protein into pT25 to obtain the pFliF-Bs1 plasmid. *Bacillus subtilis* fluorescent protein Bs1 (15) was fused to the FliF C-terminal with a 20-aa linker (N-LAEAAAKEAAAKEAAAKAAA-C). FliF-Bs1 expression was controlled by an arabinose-inducible promoter.

***Growth Conditions***

It is known that the end-to-end distance of bacterial motility and chemotaxis peaks at around 30 ℃ (16). Therefore, we used 30 ℃ in our experimental conditions. Bacterial overnight cultures were prepared in LB medium (1% tryptone, 0.5% yeast extract, 1% NaCl) with aeration at 200 rpm at 30 ℃ and used for either bacterial-host cell interactions or further experimental purposes. For Western blot and RNA-seq sample preparation, bacteria were cultured in LB medium and harvested at OD_600_=0.5 to 0.6. For motility assay, TEM analysis, and qRT-PCR sample preparation, bacteria were inoculated into 0.35% soft-agar LB plates for 24 to 60 hours. When necessary, the following chemicals were added into growth medium to the indicated final concentration: 100 μg/mL ampicillin; 50 μg/mL kanamycin; 34 μg/mL chloramphenicol; 0.1-0.2% arabinose; 0.2% pyruvate; and 0.2% glucose. For anaerobic conditions, all growth conditions were the same as for aerobic conditions except that the overnight aerobic bacterial cultures were re-inoculated in either fresh LB medium or a 0.35% soft-agar LB plate supplemented with 0.2% pyruvate and incubated in an anaerobic chamber equilibrated at 85% N_2_, 5% CO_2_, and 10% H_2_.

**Motility Assay**

Bacterial swimming motility was examined on 0.35% soft-agar LB plates (1% tryptone, 0.5% yeast extract, 1% NaCl, and 0.35% agar), as described previously (1). Plate images over time was captured using an Epson Perfection 4990 Photo scanner, and the diameters of motility zones formed on plates were measured in Fiji software version 2.3.0 (17). Statistical analysis was carried out in Prism 9 (GraphPad Software, Boston, Massachusetts USA, www.graphpad.com). Motility assays were performed with 5-20 biological repeats.

**Western Blotting**

Western blot was performed as described previously (5). In brief, bacterial pellets were resuspended in 1X sample buffer (50 mM Tris-HCl pH 6.8, 2% SDS, 12.5% glycerol, 0.001% Bromophenol blue, 2.5% 2-mercaptoethanol) and heated at 95 ℃ for 7 min. Total proteins from ~2 × 10^7^ cells were loaded onto 10 – 15% SDS-PAGE gels and resolved at 120 V in SDS running buffer. Proteins were then transferred onto 0.45 µm PVDF Immobilon-P membranes (Millipore, Burlington, MA, USA; Cat# IPVH00010) using a Mini Trans-Blot Cell system (Bio-Rad) at 400 mA for 90 min at 4 ℃ in transfer buffer (25 mM Tris pH 8.3, 192 mM glycine, 20 % methanol, and 0.1% SDS). Membranes were blocked with 6% milk in 1X TBST buffer (20 mM Tris, 150 mM NaCl, 0.05% Tween-20 pH 7.5) at 4 ℃ overnight and hybridized with individual primary antibodies. FliC was detected with anti-FliC antibody (Abcam; Cat# ab93713; RRID: AB_10563522; 1:10,000 dilution); RNase E was detected with anti-RNase E antibody (1:20,000 dilution); TolA with anti-TolA antibody (1:8000 dilution); GAPDH with anti-GAPDH antibody (SignalChem Biotech, Richmond, VA, USA; Cat# G13-61M; RRID: AB_1537483; 1:1000 dilution); FLAG-tagged proteins with Monoclonal ANTI-FLAG® M2-Peroxidase (HRP) antibody (Sigma-Aldrich; Cat# A8592; RRID: AB_439702; 1:8000 dilution); HA-tagged proteins with anti-HA antibody (1:2000 dilution); FliA proteins with anti-FliA antibody (BioLegend, Cat# 663602; RRID: AB_2566444; 1:1000 dilution); and GFP fusion proteins with anti-GFP antibody (Santa Cruz Biotechnology, Cat# sc-9996; RRID: AB_627695; 1:300 dilution). Anti-mouse or anti-rabbit secondary antibodies (Cytiva; Cat# NA931, RRID: AB_772210 and Cat# NA934; RRID: AB_772206, respectively) were used at 1:10,000 dilution. Antibody signals were detected using Amersham ECL Western Blotting Detection Reagent (Cytiva; Cat# RPN2106) or Immobilon Western Chemiluminescent HRP Substrate (Millipore; Cat# WBKLS0500) and captured using a BioSpectrum 815 system (UVP).

**Analysis of Protein Stability**

To obtain protein half-life samples, chloramphenicol was added to exponentially growing cultures (OD_600_ = 0.5–0.6) at a final concentration of 200 µg/mL (as previously described (18)) to inhibit protein synthesis (19). Bacterial samples were then harvested at 0, 0.5, 1, 2, and 4 hours after chloramphenicol addition by centrifugation at 13,000 rpm for 1 min. Total protein from these samples was subsequently subjected to Western blot analysis. Three biological replicates were performed.

**FLAG-FlgM Secretion Assay**

The FlgM secretion assay was performed as previously described (20). In brief, bacterial strains carrying pflgM plasmid (expressing FLAG-FlgM) were cultured in LB medium with appropriate antibiotics. Arabinose (0.2%) was added at OD_600_ ~0.3, and aliquots of bacterial culture were collected after 30 min of induction to examine intracellular FLAG-FlgM levels. The remaining culture was grown for another 3 h until OD_600_ ~1.0. Supernatants from 10 mL of bacterial culture at OD_600_ ~1.0 were obtained by centrifugation at 4000 × *g* for 10 min. To ensure complete removal of residual bacterial contamination, the supernatant was transferred to a new tube and centrifuged again. Proteins in the supernatant were precipitated with 15% trichloroacetic acid (TCA) on ice for 30 min, pelleted by centrifugation at 14,000 rpm for 5 min at 4 ℃, and washed twice with ice-cold acetone before resuspension in 1X SDS sample buffer. Both intracellular and extracellular (secreted) samples were resolved on 15% SDS-PAGE gels, and FLAG-FlgM abundance was analyzed by Western blotting. Three biological replicates were performed.

**RNA Isolation**

Total RNA from liquid cultures was extracted as previously described (21). Briefly, 4 mL of ice-cold stop solution (95% EtOH, 5% phenol saturated with buffer, pH 6.6–7.9) was added to 24 mL of bacterial culture to halt enzymatic activity. Bacteria were then pelleted by centrifugation at 4000 × *g* for 10 min at 4 ℃. For RNA isolation, bacterial pellets were resuspended in 2 mL of KJ medium (50 mM glucose, 25 mM Tris-HCl pH 8.0, 10 mM EDTA pH 8.0, 100 mM NaCl) and lysed by boiling in 2 mL lysis buffer (0.2 M NaCl, 20 mM Tris-HCl pH 7.5, 40 mM EDTA pH 8.0, 0.5% SDS) for 45 sec. RNA was extracted by adding 2 mL of acid phenol (pH 4.5) and mixed thoroughly by slow inversion ~20 times. The aqueous phase containing RNA was separated by centrifugation at 4000 × *g* for 1 hour at 4 ℃. Total RNA was precipitated with 1 volume of isopropanol and 1/10 volume of 3 M sodium acetate (pH 7.8) at –20 ℃ for at least 2 hours. For RNA half-life samples, rifampicin was added to the growing liquid culture at a final concentration of 500 µg/mL (from a 50 mg/mL stock) to inhibit de novo RNA synthesis (22). Aliquots of the bacterial liquid culture were collected before and after 1, 2, 4, 8, and 16 min of rifampicin treatment for RNA half-life determination.

To extract RNA from plate cultures, some modifications were made. Briefly, ~3 to 4 mL of soft agar containing bacterial cells was collected into a 50-mL Falcon tube. Bacterial enzymatic activity was stopped by incubation in 8 mL of stop solution for 3 min. The soft agar containing bacterial cells was pelleted by centrifugation at 12,000 rpm for 5 min at 4 ℃. Then, agar pellets were resuspended in 4 mL of KJ medium and 4 mL of lysis buffer. To lyse the bacteria in soft agar, the mixture was boiled in a hot water bath for 3 min. Total RNA was extracted by adding 4 mL of acid phenol (pH 4.5) and mixed thoroughly by slow inversion ~30 times. Then, total RNA was isolated in the aqueous phase as described above.

**Northern Blotting**

Northern blotting was performed as described (5). Briefly, 8 μg of total RNA was separated on a 7 M urea gel containing 6% polyacrylamide (acrylamide/bis-acrylamide 19:1) in 0.5X TBE at 120 V for 70 min, until the xylene cyanol dye reached one-quarter of the distance from the bottom of the gel. RNA was transferred onto Zeta-Probe® Blotting membranes (Bio-Rad, Hercules, CA, USA; Cat# 162-0165) at 40 mA for 90 min at 4 ℃ in 0.5X TBE buffer using a Trans-Blot Electrophoretic Transfer Cell system (Bio-Rad) and UV-crosslinked to the membrane with a Stratalinker 2400 UV Crosslinker (Stratagene) at 120,000 microjoules/cm^2^. Membranes were pre-hybridized with ULTRAhyb™-Oligo hybridization buffer (Invitrogen) for 6 h at 42 ℃. A radioactive antisense DNA oligo probe was used to detect the target RNA. Probe sequences used in this study are listed in Table S3. T4 polynucleotide kinase (NEB) was used to label the 5' end of the oligo probe with [γ-32P] ATP. Radioactive probes were then purified on a MicroSpin G-25 column (GE Healthcare) and added to the hybridization buffer for overnight hybridization with the target RNA at 42 ℃. Wash solutions I and II (2X or 0.5X SSC, respectively, with 0.1% SDS) were used to remove unbound radiolabeled oligo probe. Northern blot signals were captured using a super-resolution BAS Storage Phosphor Screen (GE Healthcare) and detected with a GE Amersham Typhoon system.

**RNA Sequencing (RNA-seq) Analysis**

Whole transcriptome gene expression profiles for cultures on soft agar plates were obtained by next-generation sequencing (NGS) analysis. Briefly, total RNA isolated from plate cultures was pelleted at 15,000 rpm for 15 min at 4 ℃, washed with 70% ethanol, and dissolved in DEPC H_2_O. DNase I (ROCHE, Cat# 04716728001) was applied to remove genomic DNA according to the manufacturer’s protocol. RNA samples (15 µg) were submitted to the Genomics Core of the Institute of Molecular Biology (IMB, Academia Sinica, Taiwan) for RNA quality assessment and RNA-seq analysis. RNA quality was evaluated using a Bioanalyzer (Agilent 2100), and only samples with an RNA Integrity Number (RIN) above 8.5 were selected for sequencing. ExFold RNA Spike-In Mixes (Thermo Fisher Scientific, Waltham, MA, USA; Cat# 4456739) were used as an internal control before rRNA depletion to monitor the entire RNA-seq process and to aid normalization, following the manufacturer’s instructions (23). Briefly, 5 µg of total RNA was mixed with 1 µl of 1:10 diluted ERCC spike-in RNA to a final volume of 50 µl. rRNA was then depleted using a RiboMinus Bacteria 2.0 Transcriptome Isolation Kit (Thermo Fisher Scientific, Waltham, MA, USA). RNA quality was verified again after rRNA depletion using a Bioanalyzer. Sequencing libraries were constructed using the TruSeq Stranded Total RNA kit (Illumina) to generate cDNA libraries with insert sizes of ~200 to 500 base pairs (bp), with adaptors of 135 bp at both ends. Briefly, total RNA was fragmented using divalent cations to generate RNA fragments of 200–500 nt during cDNA library preparation. A stranded protocol incorporating dUTPs instead of dTTPs during second-strand cDNA synthesis was used to achieve strand specificity of the cDNA library. Adaptors with unique 8-nt index sequences were added to the cDNA ends before library amplification. Twelve PCR cycles were used to amplify the cDNA library, which was further diluted to 4 nM before equal pooling for sequencing. Then, 10 µl of each library was equally pooled onto the flow cell. Sequencing was performed using a NextSeq 500 Sequencing System (Illumina; single read 75 nt) with NextSeq500 High Output 75 cycles (Illumina), according to the manufacturer’s instructions. We collected 400 million reads, resulting in ~42 GB of raw data. To filter non-specific signals, only reads with detected index primers on each sequence read were counted as proofed reads and further subjected to RNA-seq analysis. RNA sequencing analysis was performed with four biological replicates.

**RNA-seq Data Analysis**

The RNA-seq output raw data were processed by the Bioinformatics Core of the Institute of Molecular Biology (IMB, Academia Sinica, Taiwan) to generate read counts for each gene. Briefly, raw sequence reads in fastq format were mapped to the *E. coli* K-12 reference genome and the annotated transcriptome (GCF_000005845.2; ASM584v2) using STAR (v 2.7.10) alignment (24). Transcript abundances of 4,626 genes and 92 spike-in ERCC RNAs from the RNA-seq data were quantified by RSEM (v1.3.3) (25) to generate the read count of each gene. The read counts were further normalized to Transcripts Per Million (TPM) for comparative analysis. Briefly, the TPM for each gene was derived by dividing the raw read count by the gene length in kilobases to obtain RPK (reads per kilobase). Then, the sum of all RPK values in a sample was divided by 1,000,000 to obtain the "per million scaling factor." Finally, the RPK for each gene was divided by the "per million scaling factor" to obtain TPM (transcripts per million). The corresponding data have been deposited in NCBI GEO (accession# GSE282518).

Log_2_ fold change values were plotted in Prism 9 (GraphPad) to demonstrate differential up- or down-regulation of steady-state target transcripts in the Rned500 and Rned500/pFL strains relative to the parental MG1655_mv strain.

**Quantitative Reverse Transcription PCR (qRT-PCR)**

Quantitative reverse transcription polymerase chain reaction (qRT-PCR) was performed using a SuperScript™ IV First-Strand Synthesis System (Invitrogen) and iQ™ SYBR^®^ Green Supermix (Bio-Rad, Hercules, CA, USA) according to the manufacturers’ instructions. Briefly, 2.5 μg of total RNA isolated from either soft agar plate cultures (for steady-state RNA levels) or liquid cultures (for mRNA half-lives) was reverse transcribed with 50 ng of random hexamers to generate cDNA at 53 ℃ for 12 min. RNase H was then used to remove RNA. A 2.5-fold dilution of the cDNA was used for each qRT-PCR reaction, which was performed on a CFX Opus Real-Time PCR System (Bio-Rad, Hercules, CA, USA). The *mreB* gene was used as an internal reference to normalize quantifications of steady-state cDNA levels (26). The 16S rRNA gene was used as an internal reference to normalize cDNA levels across different sampling time points for mRNA half-life determination. Primers were used at a concentration of 100–250 nM to amplify target genes. The oligo primers used for qRT-PCR are listed in Table S4. The 2^-ΔΔCt method (27) was used to determine the relative expression of target transcripts at steady state or different sampling time points for half-life determination in the Rned500 strain compared to parental MG1655_mv. At least three biological replicates with triplicate technical replicates were performed for each target gene. Log_2_ values of 2^-ΔΔCt were plotted in Prism 9 (GraphPad) to demonstrate the differential up- or down-regulation of steady-state target transcripts in the Rned500 strain relative to the parental MG1655_mv strain. The qRT-PCR raw data used for plotting Figure 4B are listed in Table S5.

**Transmission Electron Microscopy (TEM)**

Flagellar filaments of bacterial cells in LB soft agar were observed by transmission electron microscopy (TEM), as previously described with modifications (28). Briefly, LB soft agar containing bacterial cultures after 24 hours of growth was taken and gently mixed with 2 volumes of 1 mM MgSO_4_ and incubated at 30 ℃ for 2 h to release the bacteria from the soft agar. Samples were loaded onto a copper grid (Ted Pella, Inc., Cat# 01753-F) and stained with 1% uranyl acetate for 25 sec. TEM images were captured using a Tecnai G2 Spirit TWIN system (Thermo Fisher Scientific). Approximately 150–200 individual bacterial cells from at least 50 individual images of three biological replicates were analyzed to calculate the percentage of flagellated/non-flagellated cells.

**Fluorescence Imaging**

For fluorescence microscopy of FliF-Bs1, bacteria were cultured in LB medium with 0.2% arabinose and harvested at OD₆₀₀ ~0.5. Cells were then washed with PBS and resuspended in one-sixth of the original volume in PBS. Fluorescence images were acquired with a Zeiss AxioObserver Z1 microscope equipped with a Prime BSI Scientific CMOS camera (PHOTOMETRICS), Plan Apochromat 63X/1.4 oil DIC objective, 495 nm and 519 nm lasers, and an EX450/20 + T470lpxr + 520/60m filter set. Images were further processed in Fiji software version 2.3.0 (17). Three biological replicates were analyzed.

**THP-1 and HEK293T Cell Culture**

Human monocytic THP-1 cells (ATCC, TIB-202) and Human Embryonic Kidney 293T (HEK293T) cells (ATCC, CRL-11268™) were cultured at 37 ℃ in a humidified 5% CO₂ atmosphere. THP-1 cells were maintained in Roswell Park Memorial Institute (RPMI) 1640 Medium (Gibco™; Cat# 11879020) supplemented with 10% Fetal Bovine Serum (FBS, Cytiva HyClone™; Cat# SH30396.03), 100 units/mL penicillin, 100 μg/mL streptomycin (Penicillin-Streptomycin, 10,000 U/mL; Gibco™; Cat# 15140148), and 50 μM β-mercaptoethanol (2-Mercaptoethanol, 50 mM; Gibco™; Cat# 31350010).

HEK293T cells were cultured in Dulbecco’s Modified Eagle Medium (DMEM) High Glucose (Gibco™; Cat# 11960077) supplemented with the same concentrations of FBS and Penicillin-Streptomycin.

**Bacterial Challenge, Immunofluorescence Labeling, and Confocal Microscopy**

To examine the ability of different *E. coli* strains to trigger NF-κB nuclear translocation, 1 × 10^5^ THP-1 cells were seeded onto 18-mm^2^ coverslips pre-coated with Poly-D-lysine hydrobromide (Sigma-Aldrich, Burlington, MA, USA; Cat# P7886) in 12-well plates. Overnight bacterial cultures, grown in LB medium at 30 ℃ (as previously described), were pelleted, resuspended in RPMI 1640 medium, and heat-killed at 65 ℃ for 20 min. The heat-killed *E. coli* was then used to challenge THP-1 cells at a multiplicity of infection (MOI) of 100 for 6 h at 37 ℃ in a humidified 5% CO_2_ atmosphere (29, 30).

To detect the intracellular distribution of NF-κB, immunofluorescence labeling was performed as previously described (31). Briefly, THP-1 cells were fixed with 4% paraformaldehyde (Electron Microscopy Sciences; Cat# 15710) in PBS for 10 min at room temperature, permeabilized with 0.1% Triton X-100 in PBS for 10 min, blocked with 1% bovine serum albumin (Bio Basic Inc., Canada; Cat# AD0023SP) in PBS for 1 h, and then incubated with NF-κB polyclonal p65 primary antibody (1:100 dilution; Cell Signaling Technology; Cat# 8242; RRID: AB_10859369) overnight at 4 ℃. After washing with PBS, cells were labeled with Goat anti-Rabbit IgG (H+L) Cross-Adsorbed Secondary Antibody, Alexa Fluor™ 488 (1:1000 dilution; Invitrogen™; Cat# A-11008; RRID: AB_143165) for 1 h at room temperature. Nuclei were stained with Hoechst 33342 (Thermo Fisher Scientific, Waltham, MA, USA; Cat# 62249) for 5 min at room temperature before mounting with mounting medium (Vector Laboratories).

Images of NF-κB localization and stained nuclei at the middle Z-section were acquired using either an LSM710 microscope (Carl Zeiss, Inc.) or a high-content screening microscope system (ImageXpress Micro XL System, Molecular Devices) with a 63X or 20X objective, respectively. The NF-κB nuclear-to-cytoplasmic ratio was determined as previously described (32). In brief, Hoechst signal defined the nuclear boundary, and brightfield images delineated the cell boundary. NF-κB intensity in the nucleus and entire cell was measured using Fiji software (version 2.3.0) (17). Cytosolic intensity was calculated by subtracting nuclear intensity from total cellular intensity, and the NF-κB nuclear:cytoplasmic ratio was determined by dividing nuclear intensity by cytosolic intensity. Statistical analysis was performed on 35 individual cells from at least five independent immunofluorescence images of three biological repeats.

**Plasmids, Co-transfection of Plasmids into HEK293T Cells, and Detection of Bacterial/Flagellin TLR5-Dependent NF-κB Signaling Pathway Activation in HEK293T Cells**

For TLR5 dual luciferase reporter assay, 1 × 10^5^ cells of Human Embryonic Kidney 293T cells were seeded in a 24-well flat-bottomed polystyrene (PS) tissue culture dish (CoStar®; Cat# 38017). After 20-24 h, HEK293T cells at 50 – 60% confluency were transiently co-transfected with the following plasmids by Lipofectamine™ 3000 (Invitrogen™; Cat# L3000015), as described in the manufacturer's protocol: 10 ng of pGL3-ELAM-Luc plasmid (Addgene plasmid #13029; http://n2t.net/addgene:13029; RRID:Addgene_13029) carrying firefly luciferase under the control of the ELAM-1 (endothelial cell-leukocyte adhesion molecule 1; also known as E-selectin) proximal promoter that contains three NF-κB repeated transcription factor binding sites (33); and 1 ng of pRL-TK plasmid (Promega; Cat# E2241) carrying the *Renilla* luciferase reporter under the control of a constitutively-expressed thymidine kinase (TK) promoter (34). Firefly luciferase was used as a reporter of TLR5 – NF-κB signaling transduction. *Renilla* luciferase was used as the internal control of transfection efficiency and to ensure proper interpretation of normalized results. For expression of TLR5, 100 ng of the hTLR5 Flag plasmid (Addgene plasmid #13088; http://n2t.net/addgene:13088; RRID:Addgene_13088) was included in transient co-transfection to obtain HEK293T/TLR5 cells. For a negative control of hTLR5 Flag plasmid, we used 100 ng of pcDNA6.0 empty vector (Invitrogen™; Cat# V222-20) as a substitute to obtain HEK293T/Control cells. After 24 hours of co-transfection, the transfected HEK293T/TLR5 or HEK293T/Control cells were challenged with either purified *S. typhimurium* flagellin (FLA-ST, InvivoGen; Cat# tlrl-stfla) at a final amount of 0, 100, 200, 400 or 800 ng or with different bacterial strains at MOI = 100 as previously described with some modifications (30). The bacteria used in this reporter assay experiment were prepared from overnight culture in LB medium at 30 ℃ and 200 rpm, and resuspended in DMEM with 10% FBS and 1% of PS solution for further use. TLR5 activation was monitored using a dual-luciferase assay (35).

In brief, different amounts of purified *S. typhimurium* flagellin or MOI = 100 of different MG1655_mv derivative bacterial strains were added into either HEK293T/TLR5 or HEK293T/Control cell culture and incubated for 6 h at 37 ℃ and 5% CO_2_. The MG1655_mvΔ*fliC* strain that does not express flagellin was used as a negative control for bacterial challenge. To lyse cells for luciferase assay, the HEK239T cells were first washed with PBS buffer and lysed with 100 μl of passive lysis buffer (Promega; Cat# E1980) at room temperature for 15 min according to the instruction manual. After lysing cells, 20 μl of the cell lysate was transferred to a 96-well flat-bottom white polystyrene plate (Costar; Cat# 3917) and NF-κB activation was determined as the ratio of firefly to *Renilla* luciferase light emission by deploying a Dual-Luciferase assay kit (Promega; Cat# E1980). The firefly and *Renilla* luciferase light emissions were obtained sequentially using a VICTOR3 Multilabel Plate Reader (PerkinElmer). In brief, 100 μl of Luciferase Assay Buffer II that contained Luciferase Assay Substrate was added into each well. After a 2-s delay, firefly light emission was captured for 10 s to obtain the firefly relative light units (RLU). Then, 100 μl of Stop & Glo reagent was added into each well to quench the firefly signal and activate simultaneously the *Renilla* luciferase. After a 2-s delay, *Renilla* light emission was captured for 10 s to obtain the *Renilla* relative light units (RLU). The firefly RLU of each experimental condition from either HEK293T/TLR5 or HEK293T/Control cells was first normalized to *Renilla* RLU, then further adjusted against the values from either HEK293T/TLR5 or HEK293T/Control cells treated with 0 ng flagellin or the MG1655_mvΔ*fliC* strain to obtain the relative firefly/*Renilla* luciferase activity of each experimental condition. Six experimental repeats were performed for the commercial flagellin treatment, whereas seven biological repeats with triplicate technical repeats were performed for the bacterial challenge experiments. The relative firefly/*Renilla* luciferase activity was plotted in GraphPad Prism 9, GraphPad Software, Boston, Massachusetts USA, [www.graphpad.com](http://www.graphpad.com).

**RNase E Amino Acid Motif Prediction and Alignment**

Amino acid sequences of *E. coli* (NCBI Protein: NP_415602.1), *Salmonella typhi* (NCBI Protein: WP_000827472.1), *Vibrio cholerae* (NCBI Protein: WP_046126928.1), *Pseudomonas putida* (NCBI Protein: NP_744060.1), *Bordetella pertussis* (NCBI Protein: WP_014905943.1), and *Legionella pneumophila* (NCBI Protein: WP_015444149.1) RNase E were obtained from EcoCyc (https://ecocyc.org/). Conservation and alignment of RNase E amino acid motifs from these bacterial species were predicted by MEME (Multiple Em for Motif Elicitation) Suite (<https://meme-suite.org/meme/tools/meme>) (36) by setting the number of motifs to 15 and the motif width range from 30 to 300 aa. Conservation was calculated by aligning and comparing the similarity of either the N-terminal or C-terminal region of *E. coli* RNase E. Alignment of amino acid residues between *E. coli* and *Legionella* RNase E homologs was performed using UniProt (https://www.uniprot.org/align).

**Quantification and Statistical Analysis**

Signal intensity of Western blot or fluorescence microscopy images was quantitated using Fiji software version 2.3.0 (17). Lanes showing no detectable signal above background were designated as 'Not Detected (ND)', indicating complete absence of measurable protein. Faint bands visually identifiable but yielding normalized relative abundance values consistently below our established limit of quantification (LOQ < 0.05 relative to the control) were designated 'Not Available (NA)'. Statistical significance was determined using Prism 9 (GraphPad Software, Boston, MA, USA, www.graphpad.com). Unpaired Student’s t-tests were used for comparisons between two groups. For comparisons involving three or more groups, Analysis of Variance (ANOVA) was performed. Specifically, one-way ANOVA followed by Dunnett’s post-hoc test against a control group was used when comparing multiple groups to a single control (e.g., Figure 2D and 2H). For other multiple group comparisons (e.g., Figure 3Ab), one-way ANOVA was followed by Tukey's Honestly Significant Difference (HSD) post-hoc test. Two-way Analysis of Variance (ANOVA) was used for experiments involving two independent variables (e.g., Figure 3Bb), with post-hoc comparisons for significant effects conducted using Tukey's HSD test. A p-value of < 0.05 was considered statistically significant.

**Supporting Figures and Tables**


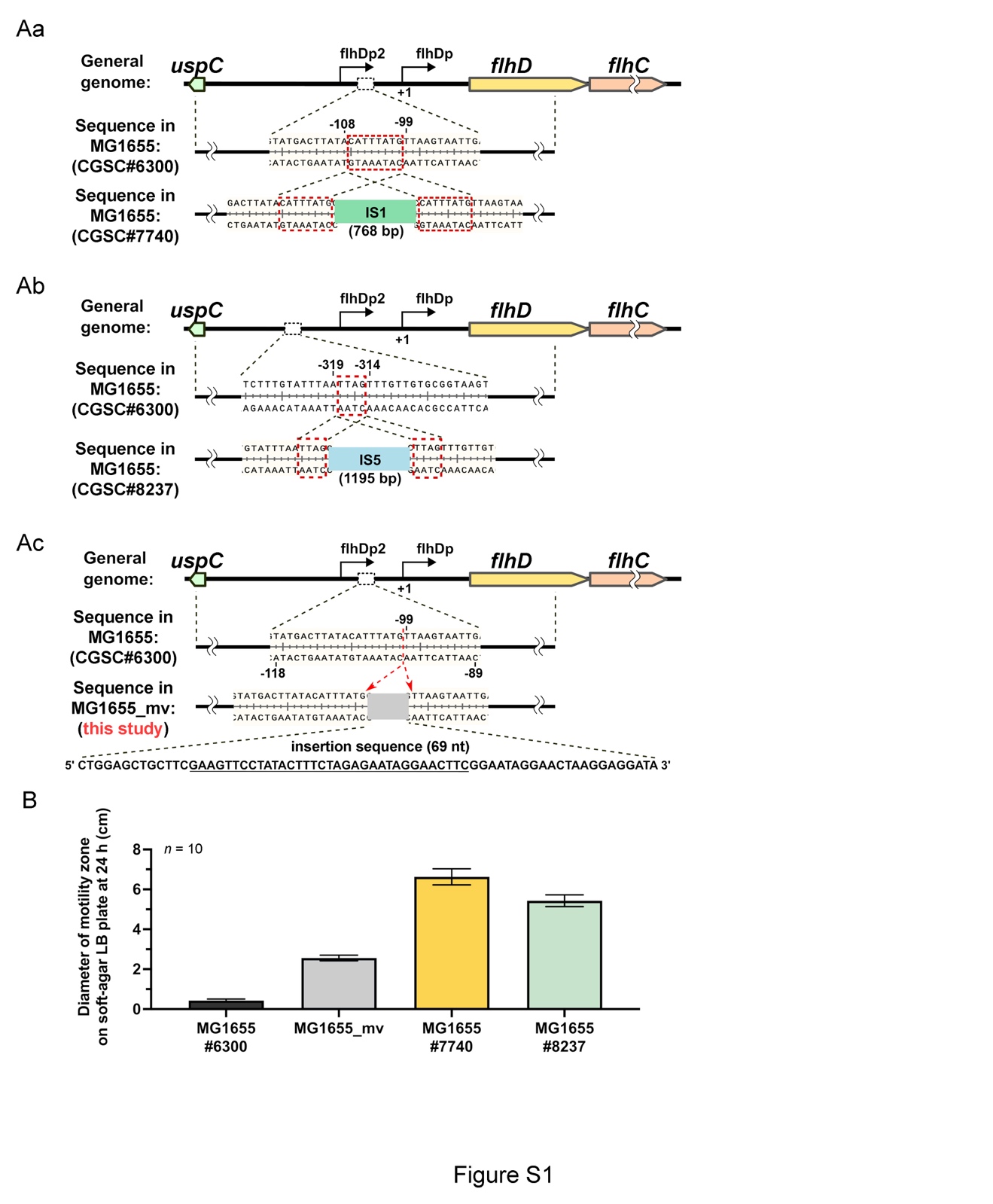


**Fig. S1.** Variations in the flhDC Promoter Region and Corresponding Motility Differences in MG1655 Strain. (Aa-Ac) Schematic representation of the chromosomal region between the flagellar class I *flhDC* genes and the upstream *uspC* gene in MG1655 (CGSC#7740) (Aa), MG1655 (CGSC#8237) (Ab), and MG1655_mv (Ac) strains compared to that from MG1655 (CGSC#6300). The *flhDp* and *fhlDp2* promoters are indicated. Nucleotide sequences from MG1655 (CGSC#6300), MG1655_mv, MG1655 (CGSC#7740), and MG1655 (CGSC#8237) strains are shown, with numbers indicating nucleotide positions upstream (–) relative to the transcription start site (+1). Red dashed boxes, terminal inverted repeats of the insertion sequence (IS) elements; red dashed line, the insertion site of the 69-nucleotide insertion sequence (shown below the scheme) in the original MG1655 (CGSC#6300) sequence. (B) Motility zone diameters of MG1655 (CGSC#6300), MG1655_mv, MG1655 (CGSC#7740), and MG1655 (CGSC#8237) strains grown on LB soft-agar plates after 24 hours of incubation. Values are mean ±SD (*n* = 10 biological replicates).


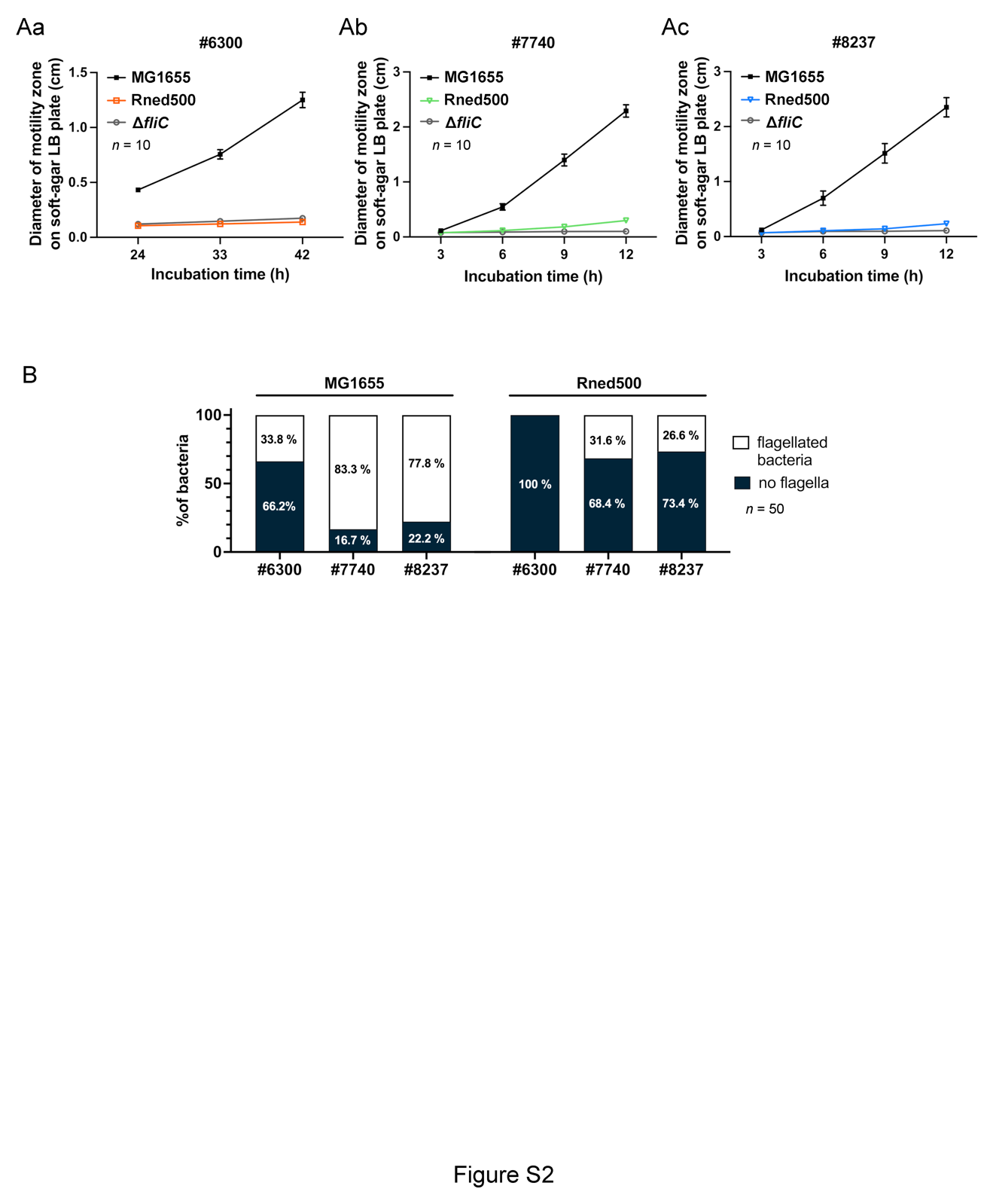


**Fig. S2.** RNase E C-terminal Truncation of the Rned500 Abolishes Motility and Reduces Flagellation Independently of *flhDC* Promoter Variation. (Aa-Ac) Motility zone diameters of MG1655 and Rned500 strains in CGSC#6300 (Aa), CGSC#7740 (Ab), and CGSC#8237 (Ac) variants grown on LB soft-agar plates for 24 hours. Values are mean ±SD (*n* = 10 biological replicates). (B) Quantification of flagellated/non-flagellated cells from TEM images of MG1655 and Rned500 strains in CGSC#6300, CGSC#7740, and CGSC#8237 variants (*n* = 50 cells/strain).


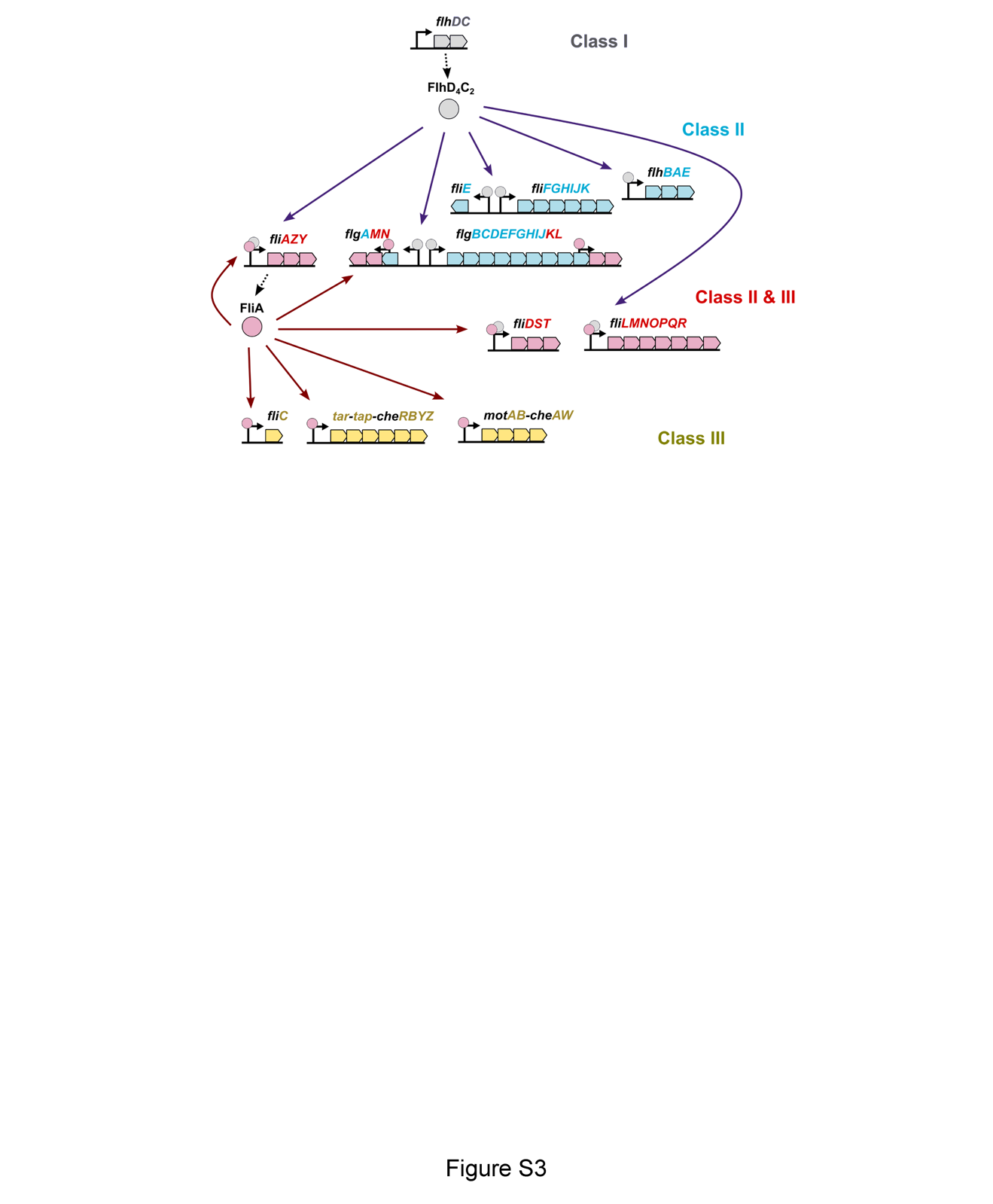


**Fig. S3.** Schematic of the Flagellar Transcriptional Cascade. Expression of 50 flagella-associated genes is organized into a three-tiered transcriptional cascade. Gray boxes, class I genes; blue boxes, class II genes; pink boxes, class II and III genes; yellow boxes, class III genes; dashed arrows, expression of the encoded protein; solid arrows, transcriptional control by FlhD_4_C_2_ or FliA.


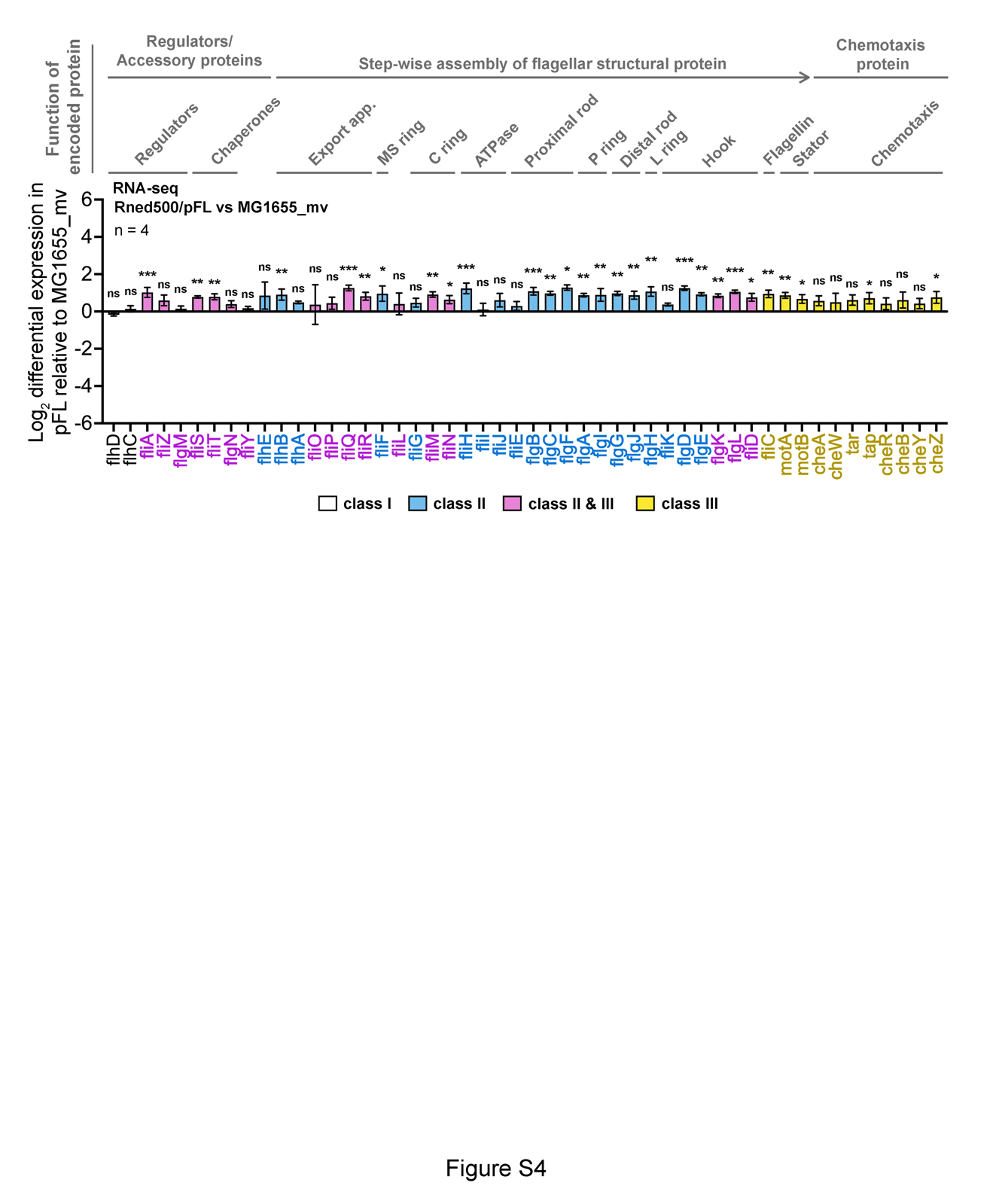


**Fig. S4.** Restoration of Flagellar Gene Expression in Rned500/pFL Strain. Differential mRNA levels in the Rned500/pFL strain compared to MG1655_mv, as determined by RNA-seq analysis. Genes are arranged according to the function of their encoded proteins, as described above the figure. Values are mean ±SD of log_2_ relative expression fold-change (*n* = 4 biological replicates; multiple unpaired *t*-test). ****P* < 0.001; ***P* < 0.01; **P* < 0.05; and ns, *P* ≥ 0.05.


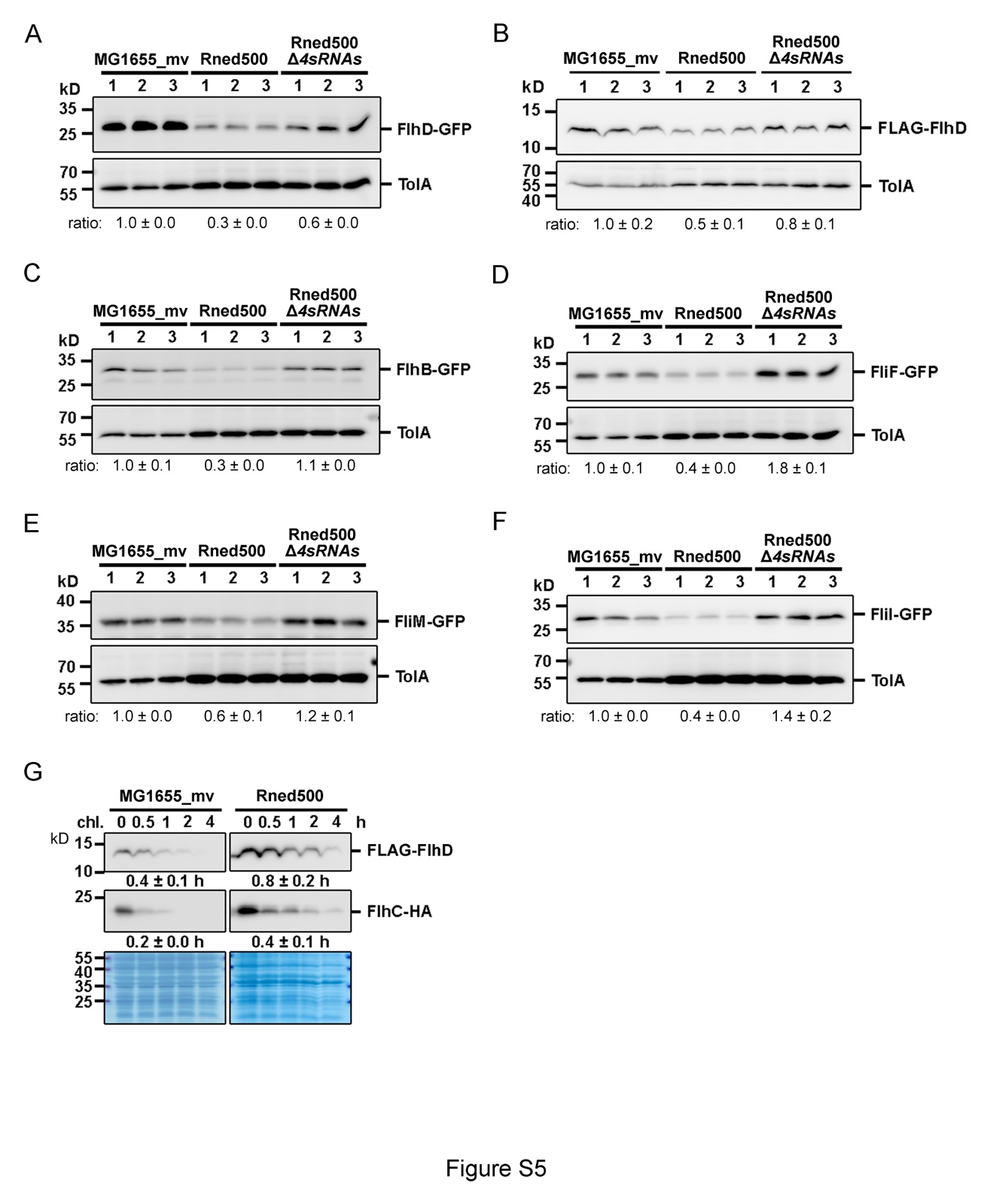


**Fig. S5.** Western Blot of Relative Levels of Flagellar GFP Fusion Proteins and FLAG-FlhD, and FlhDC Protein Stability. (A-F) Western blot of FlhD-GFP (A), FLAG-FlhD (B), FlhB-GFP (C), FliF-GFP (D), FliM-GFP (E), FliI-GFP (F) and TolA (loading control) in MG1655_mv, Rned500, and Rned500Δ*4sRNAs* strains. Antibody against GFP and TolA were used. Protein markers are indicated in kilodaltons (kD). Relative GFP fusion protein levels are shown. Values are mean ±SD (*n* = 3 replicates). (G) Western blot of FLAG-FlhD and FlhC-HA protein levels at various time points after chloramphenicol (chl.) treatment in MG1655_mv and Rned500 strains. Antibody against FLAG-tag, and HA-tag were used. Total protein integrity was confirmed by amido black staining of the membrane. Protein markers are indicated in kilodaltons (kD). Relative FLAG-FlhD and FlhC-HA abundances are as indicated. Values are mean ±SD (*n* = 4 replicates).


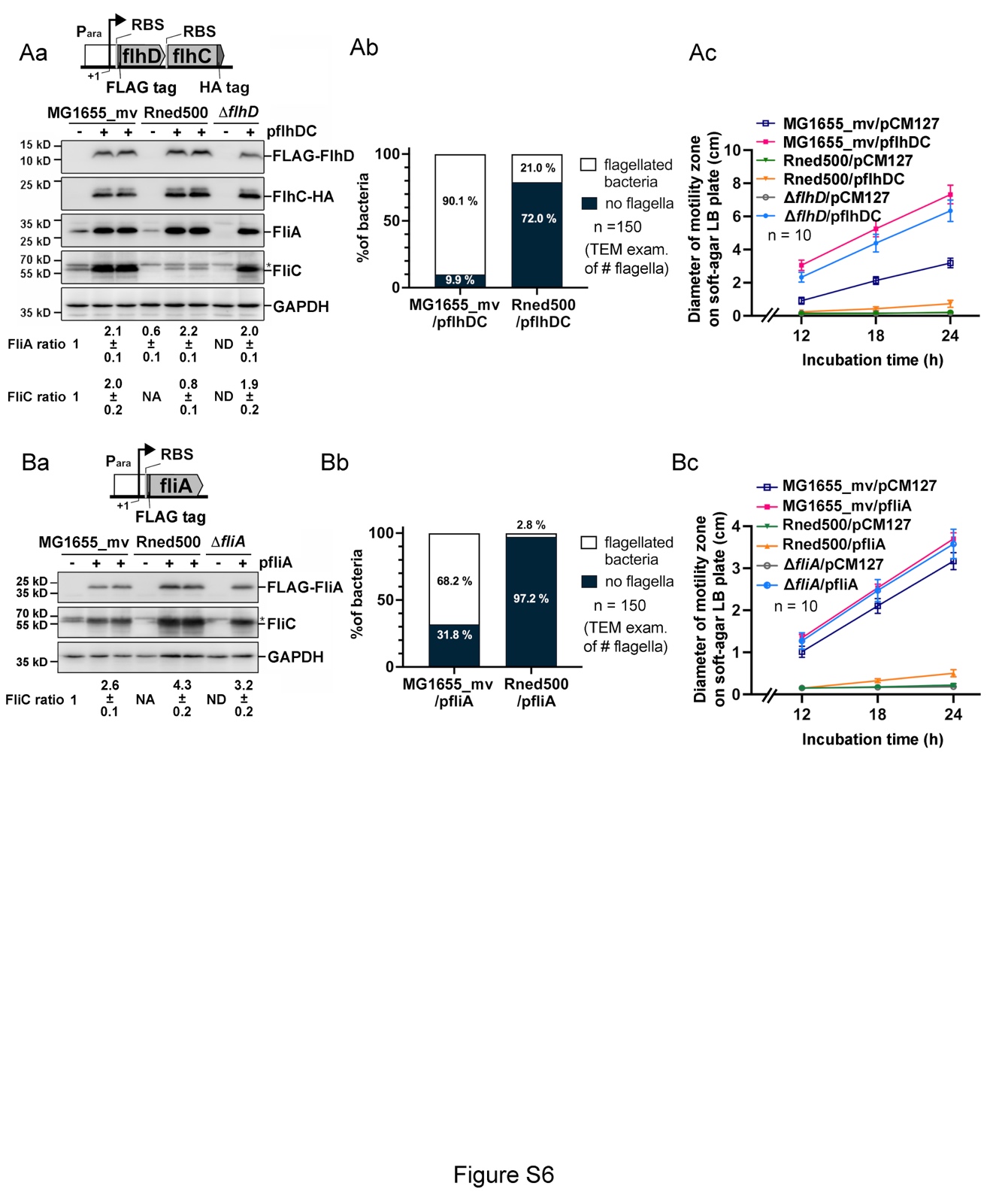


**Fig. S6.** Ectopic Expression of Class I FlhDC or Class II FliA Fails to Fully Restore Flagella Formation and Bacterial Motility in Rned500. (Aa, and Ba) Western blot of FLAG-FlhD, FlhC-HA, FliA and FliC (Aa), or FLAG-FliA and FliC (Ba) protein levels in MG1655_mv and Rned500 strains, as well as MG1655_mvΔ*flhD* or MG1655_mvΔ*fliA* control strains. Strains with pflhDC or pfliA (+) and pCM127 (−) plasmids are as indicated. The pCM127, an empty vector plasmid, was used as a negative control for the pflhDC and pfliA plasmids. GAPDH was used as loading control. Antibody against FLAG-tag, HA-tag, FliA, FliC and GAPDH were used. Asterisk, the non-specific binding signal of FliC antibody. Protein markers are indicated in kilodaltons (kD). Relative FliC protein levels are shown. Values are mean ±SD (*n* = 4 replicates). NA, not available; ND, not detected. A schematic diagram of the expression plasmids is shown above the Western blot. (Ab and Bb) Quantification of flagellated/non-flagellated cells from TEM images of FlhD/FlhC-overexpressing strains (Ab) and FliA-overexpressing strains (Bb) (*n* = 150 cells/strain). (Ac and Bc) Motility zone diameters of FlhDC-overexpressing strains (Ac) and FliA-overexpressing strains (Bc) on LB soft-agar supplemented with 0.1% arabinose for FlhDC or FliA induction over 24 h incubation. Values are mean ±SD (*n* = 10 biological replicates).

**
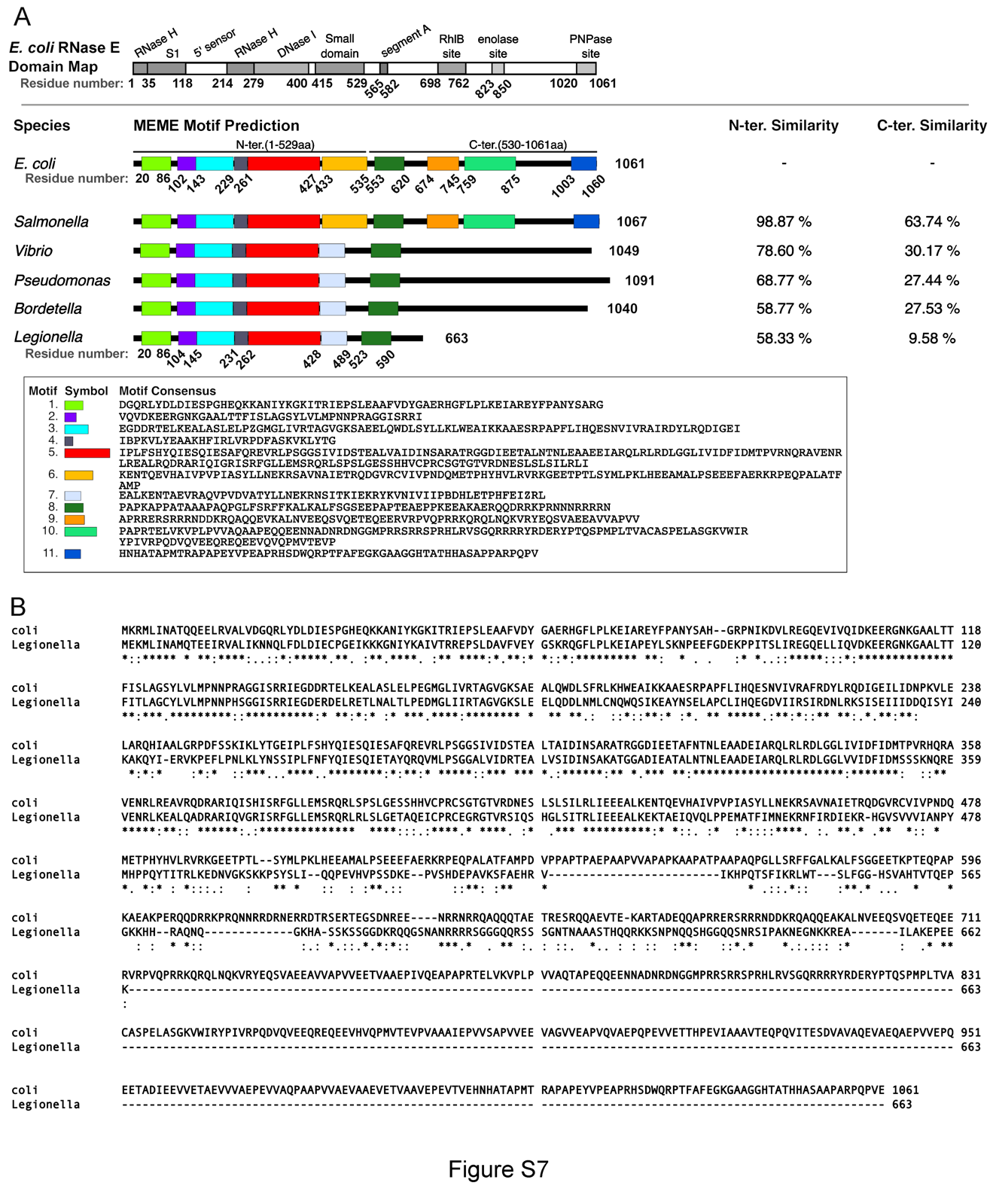
**

**Fig. S7.** Motifs Comparison of RNase E Homologs in *E. coli* and Common Human Pathogens. (A) Motifs of RNase E homologs, predicted by MEME Suite,(36) are shown in various colors. The predicted conserved amino acid sequences for each motif are listed below the panel. The linear structure of *E. coli* RNase E is depicted at the top, with labeled domains.(37) Residue numbers corresponding to each domain/motif are indicated below. The total lengths of RNase E homologs are indicated. The percentage similarity of amino acid sequences in the N-terminal and C-terminal domains, compared to the laboratory *E. coli* K-12 strain, is shown on the right. (B) Amino acid sequence alignment of *E. coli* and *Legionella* RNase E homologs. The symbols indicate: “*” conserved sequence, “:” conserved substitutions, “.” semi-conserved substitutions (similar amino acid shape), “( )” non-conserved residues, “-” gap.

Table S1. *E. coli* strains and plasmids used in this study

| ***E. coli* Strains** | **Genotype/Description** | **Antibiotic**  **Resistance** | **Source/**  **Reference** |
| --- | --- | --- | --- |
| KSL2010 | *lacZ43, relA, spoT, thi-1*, *rne*::cat *recA*::Tn*10* [pBAD-RNG] | Cm^R^/Km^R^ | Tamura *et al.* |
| KSL2010* | KSL2010 strain but pga-RNG under GAPDH promoter. | Cm^R^/Km^R^ | This paper^a^ |
| MG1655 (CGSC#6300) | K-12, *F*^-^, λ^-^, *ilvG*^-^, *rfb-50*, *rph-1* | - | Blattner *et al.* |
| MG1655 (CGSC#7740) | K-12, *F*^-^, λ^-^, *ilvG*^-^, *rfb-50*, *rph-1* | - | Coli Genetic Stock Center (CGSC) |
| MG1655 (CGSC#8237) | K-12, *F*^-^, λ^-^, *ilvG*^-^, *rfb-50*, *rph-1* | - | Coli Genetic Stock Center (CGSC) |
| MG1655_mv | isogenic variant of CGSC#6300 with 69-bp insertion at upstream of *flhDC* promoter region | - | This paper |
| Rned500–1061 | Derivative of MG1655 (CGSC#6300) strain. | Km^R^ | Murashko and Lin-Chao |
| Rned500 | Mutation moved by P1 transduction from Rned500–1061 into MG1655_mv | Km^R^ | This paper |
| CGSC#7740_Rned500 | Mutation moved by P1 transduction from Rned500–1061 into CGSC#7740 | Km^R^ | This paper |
| CGSC#8237_Rned500 | Mutation moved by P1 transduction from Rned500–1061 into CGSC#8237 | Km^R^ | This paper |
| Rned698 | Derivative of MG1655_mv. Chromosomal deletion of coding region of RhlB binding site of RNase E from 698-762 aa in MG1655_mv. | Km^R^ | This paper |
| Rned823-850 | Derivative of MG1655 (CGSC#6300) strain. Chromosomal deletion of coding region of enolase binding site of RNase E from 823-850 aa in MG1655. | Km^R^ | Murashko and Lin-Chao |
| Rned823 | Mutation moved by P1 transduction from Rned823–850 into MG1655_mv. | Km^R^ | This paper |
| Rned984-1011 | Derivative of MG1655 (CGSC#6300) strain. Chromosomal deletion of 28 aa segment of RNase E from 984-1011 aa in MG1655. Alternative control for Rned823-850. | Km^R^ | Murashko and Lin-Chao |
| Rned984 | Mutation moved by P1 transduction from Rned984–1011 into MG1655_mv. | Km^R^ | This paper |
| Rned1039 | Derivative of MG1655_mv. Chromosomal deletion of coding region of PNPase binding site of RNase E from 1039-1061 aa in MG1655_mv. | Km^R^ | This paper |
| MG1655_mvΔ*rng* | P1 transduction of *rng*::Km allele from Keio Collection into MG1655_mv. | Km^R^ | This paper |
| MG1655_mvΔ*flhD* | P1 transduction of *flhD*::Km allele from Keio Collection into MG1655_mv. | Km^R^ | This paper |
| MG1655_mvΔ*fliA* | P1 transduction of *fliA*::Km allele from Keio Collection into MG1655_mv. | Km^R^ | This paper |
| MG1655_mvΔ*fliC* | P1 transduction of *fliC*::Km allele from Keio Collection into MG1655_mv. | Km^R^ | This paper |
| MG1655_6300Δ*fliC* | P1 transduction of *fliC*::Km allele from Keio Collection into CGSC#6300. | Km^R^ | This paper |
| MG1655_7740Δ*fliC* | P1 transduction of *fliC*::Km allele from Keio Collection into CGSC#7740. | Km^R^ | This paper |
| MG1655_8237Δ*fliC* | P1 transduction of *fliC*::Km allele from Keio Collection into CGSC#8237. | Km^R^ | This paper |
| MG1655_mvΔ*fliF* | P1 transduction of *fliF*::Km allele from Keio Collection into MG1655_mv. | Km^R^ | This paper |
| MG1655_mvΔ*oxyS*Δ*arcZ*Δ*omrA*Δ*omrB*  (MG1655_mvΔ*4sRNAs*) | Derivative of MG1655_mv. *fliF*::Km. | Km^R^ | This paper |
| Rned500Δ*oxyS*Δ*arcZ*Δ*omrA*Δ*omrB*  (Rned500Δ*4sRNAs*) | Derivative of Rned500. | Km^R^ | This paper |
| **Plasmids** | **Description** | **Antibiotic**  **Resistance** | **Source/**  **Reference** |
| pBAD-EBFP2 | pBR322 *ori*, Ap^R^ | Ap^R^ | Addgene  Plasmid #14891 |
| pBAD | pBAD-EBFP2 digested with NdeI and EcoRI followed by self-ligation. | Ap^R^ | This paper |
| pBAD-Rnewt-Km | pBR322 *ori,* derived from pBAD-EBFP2. Template for pBAD-Rned698-762-Km and pBAD-Rned1039-1061-Km plasmid construction. | Ap^R^/Km^R^ | Murashko and Lin-Chao |
| pBAD-Rned698-762-Km | pBR322 *ori,* derived from pBAD-EBFP2. Template for Rned698-762 Km^R^ mutant construction. pBAD-Rnewt-Km was re-amplified with d762_fw/d698_rev and self-ligated. | Ap^R^/Km^R^ | This paper |
| pBAD-Rned1039-1061-Km | pBR322 *ori,* derived from pBAD-EBFP2. Template for Rned1039-1061 Km^R^ mutant construction. pBAD-Rnewt-Km was re-amplified with d1061_fw/d1039_rev and self-ligated. | Ap^R^/Km^R^ | This paper |
| pBAD-RNE (pRNE) | pBR322 *ori,* derived from pBAD-EBFP2. *rne* under arabinose promoter. *rne* gene was inserted between *NdeI* and *EcoRI.* | Ap^R^ | This paper |
| pBAD-DM (pDM) | pBR322 *ori,* derived from pBAD-EBFP2. *rne* D303N, D346N double mutations under arabinose promoter. *rne* gene was inserted between *NdeI* and *EcoRI.* | Ap^R^ | This paper |
| pCM128 | pSC101 *ori* Ap^r^, Par^+^ derivative of pPM28 | Ap^R^ | Tucker *et al.* |
| pCM127 | pSC101 *ori*, derived from pCM128. | Ap^R^ | This paper |
| pCM-flagRNE (pFL) | pSC101 *ori*, derived from pCM128. N-terminal FLAG-tagged RNase E is driven by *rne* native promoter and terminated with its own terminator. *rne* gene was inserted between *EcoRI* and *BamHI.* | Ap^R^ | This paper |
| pCM-flagRned500 (p500) | pSC101 *ori*, derived from pFL with *rne* gene only encodes 1-499 aa. | Ap^R^ | This paper |
| pCM-flagCter500 (pCter500) | pSC101 *ori*, derived from pFL with *rne* gene only encodes 500-1061 aa. | Ap^R^ | This paper |
| pCM-Para-FLAG-flhDC-HA (pflhDC) | pSC101 *ori*, derived from pCM128. Arabinose induced N-terminal FLAG-tagged FlhD and C-terminal HA-tagged FlhC was encoded. | Ap^R^ | This paper |
| pCM-PflhD-FLAG-flhD (pPflhD) | pSC101 *ori*, derived from pCM128. N-terminal FLAG-tagged FlhD was under the control of *flhDC* promoter. | Ap^R^ | This paper |
| pCM-Para-FLAG-fliA (pfliA) | pSC101 *ori*, derived from pCM128. Arabinose induced N-terminal FLAG-tagged FliA was encoded. | Ap^R^ | This paper |
| pCM-Para-FLAG-flgM (pflgM) | pSC101 *ori*, derived from pCM128. Arabinose induced N-terminal FLAG-tagged FlgM was encoded. | Ap^R^ | This paper |
| pga-RNG | pSC101 *ori*, derived from pBAD-RNG. RNase G is driven by GAPDH promoter. | Km^R^ | This study |
| pT25 | p15A *ori*. | Cm^R^ | Karimova *et al*. |
| pflhD-GFP | p15A *ori*, derived from pT25. Coding region from -678 to +188 nt of *flhD* gene that contain the promoter region and 30 aa FlhD coding region was cloned in frame with GFP to generate translational fusion reporter. FlhD-GFP was under the control of *flhD* promoter. | Cm^R^ | This paper |
| pfliF-GFP | p15A *ori*, derived from pT25. Coding region from -280 to +114 nt of *fliF* gene that contains the promoter region and 30 aa FliF coding region was cloned in frame with GFP to generate translational fusion reporter. FliF-GFP was under the control of *fliF* promoter. | Cm^R^ | This paper |
| pfliM-GFP | p15A *ori*, derived from pT25. Coding region from -1073 to +127 nt of *flgM* gene that contain the promoter region and 30 aa FlgM coding region was cloned in frame with GFP to generate translational fusion reporter. FlgM-GFP was under the control of *flgM* promoter. | Cm^R^ | This paper |
| pflhB-GFP | p15A *ori*, derived from pT25. Coding region from -532 to +115 nt of *flhB* gene that contain the promoter region and 30 aa FlhB coding region was cloned in frame with GFP to generate translational fusion reporter. FlhB-GFP was under the control of *flhB* promoter. | Cm^R^ | This paper |
| pfliI-GFP | p15A *ori*, derived from pT25. Coding region from -280 to +3427 nt of *fliFGHI* operon that contain the promoter region and 26 aa FliI coding region was cloned in frame with GFP to generate translational fusion reporter. FliI-GFP was under the control of *fliFGHI* promoter. | Cm^R^ | This paper |
| pFliF-Bs1 | p15A *ori*, derived from pT25. FliF-linker-Bs1 fusion protein was driven by arabinose promoter. | Cm^R^ | This paper |
| pcDNA6.0 | Cat# V222-20 | Ap^R^ | Invitrogen |
| hTLR5 flag | Addgene Plasmid #13088 | Ap^R^ | Chang *et al*. |
| pGL3-ELAM-luc | Addgene Plasmid #13029 | Ap^R^ | Chang *et al*. |
| pRL-TK | Cat# E2241 | Ap^R^ | Promega |

^a^: KSL2010* strain was constructed by S.-K. Chen (Taipei Medical University, Taipei 11529, Taiwan).

Table S2. Primers for strain and plasmid construction

| **Primer Name** | **Sequence (5' – 3')** | **Purpose** |
| --- | --- | --- |
| IS ex F | GAA ATA CAC CCA AAA CAA AAG TAT GAC TTA TAC ATT TAT GCT GGA GCT GCT TCG AAG TTC | MG1655_mv construction |
| IS ex R | AAT GCG TGA TGC AGA TCA CAC AAA ACA CTC AAT TAC TTA ATA TCC TCC TTA GTT CCT ATT | MG1655_mv construction |
| d762_fw | P-ACA GAA CTG GTG AAA GTC CC | Rned698 construction |
| d698_rw | P-CAG CGC CTT CGC TTC TTG T | Rned698 construction |
| d1061_fw | P-TAA GAA TTC GAT TCT ATT CCG AAG TTC CTA | Rned1039 construction |
| d1039_rw | P-TTT ACC TTC GAA GGC AAA AG | Rned1039 construction |
| GR_rne_fw | CGC AGC TTA GTC GTC AAT GTA AGA ATA ATG AGT AAG TTA CGA TGA AAA GAA TGT TAA TC | *rne* mutant construction |
| GR_rne_rev | GCC CTG GCA GTT ACC AGG GCT TGA TTA CTT TGA GCT AAT TAG AAT TCA CTA GTG ATT TG | *rne* mutant construction |
| OxyS del F | GAA ACG GAG CGG CAC CTC TTT TAA CCC TTG AAG TCA CTG Cct gga gct gct tcg aag ttc | Construction of *oxyS* deletion |
| OxyS del R | AAA AAA AAG CGG ATC CTG GAG ATC CGC AAA AGT TCA CGT Tta tcc tcc tta gtt cct att | Construction of *oxyS* deletion |
| arcZ del F | GTG CGG CCT GAA AAA CAG TGC TGT GCC CTT GTA ACT CAT Cct gga gct gct tcg aag ttc | Construction of *arcZ* deletion |
| arcZ del R | CGG CTA GAC CGG GGT GCG CGA ATA CTG CGC CAA CAC CAG Gta tcc tcc tta gtt cct att | Construction of *arcZ* deletion |
| omrB del F | CCC AGA GGT ATT GAT AGG TGA AGT CAA CTT CGG GTT GAG Cct gga gct gct tcg aag ttc | Construction of *omrB* deletion |
| omrB del R | AAA AAA AAC CTG CGC ATC TGC GCA GGC TGG TGT AAT TCA Tta tcc tcc tta gtt cct att | Construction of *omrB* deletion |
| omrA del F | CCC AGA GGT ATT GAT TGG TGA GAT TAT TCG GTA CGC TCT Tct gga gct gct tcg aag ttc | Construction of *omrA* deletion |
| omrA del R | AAA AAA AAC CTG CGC ATC CGC GCA GGT TGG TGC AAG AGA Cta tcc tcc tta gtt cct att | Construction of *omrA* deletion |
| RNE 600 up | P-TTT TTA CGG ATG GAG TCT CTG TTT TCA TGG | pCM-flagRNE construction |
| pi-RNE_280 RW | P-ACA TCA TGC AGC AAA CGG C | pCM-flagRNE construction |
| Add_flag_to_Rne | P-GAC TAC AAA GAC GAT GAC GAT AAA atg AAA AGA ATG TTA ATC AA | pCM-flagRNE construction |
| RNE promo R | P-CAT CGT AAC TTA CTC ATT ATT CTT ACA TTG ACG | pCM-flagRNE construction |
| Rne499-P-Re | P-GCT TAA GGT TGG GGT TTC TTC CCC T | Construct pCM-flagRned500 |
| RNE 3UTR F | TAA TTA GCT CAA AGT AAT CAA GCC CTA GTA AC | Construct pCM-flagRned500 |
| Rne600 re | P-CGC TTC TGC TTT CGG TGC TGG TTG CTC GGT | Construct pCM-flagRned500 |
| C-ter500 F | P-TAC ATG CTG CCG AAG | Construct pCM-flagCter500 |
| C-ter500 R | P-TTT ATC GTC ATC GTC TTT GT | Construct pCM-flagCter500 |
| C-ter600 F | P-AAACCGGAACGTCAACAGG | Construct pCM-flagCter600 |
| NdeI_Rne_Fw | GGA ATT CCA TAT GAA AAG AAT GTT AAT CAA CGC | pBAD-RNE construction |
| EcoRI-rne-2 rev | CGG AAT TCT TAC TCA ACA GGT TGC GGA CGC | pBAD-RNE construction |
| D346N F | P-GTT ATC AAC TTC ATC GAC ATG ACG CCA GTA CGC | pBAD-RNE_DM_ construction |
| D346N R | P-TGT CGA TGA AGT TGA TAA CAA TCA GGC CGC CGA G | pBAD-RNE_DM_ construction |
| D303N F | AAC GGC CAT CAA CAT CAA CTC CGC ACG CGC GAC CC | pBAD-RNE_DM_ construction |
| D303N R | CGG AGT TGA TGT TGA TGG CCG TTA ACG CTT CGG TG | pBAD-RNE_DM_ construction |
| flhDC F | P-ATG CAT ACC TCC GAG | pCM-P*_ara_*-flag-flhDC-HA construction |
| flhDC R | P-TTA AAC AGC CTG TAC TC | pCM-P*_ara_*-flag-flhDC-HA construction |
| flhC_HA F | P-CGA TGT TCC AGA TTA CGC TTA ATA AGA ATT CGA AGC TTG G | pCM-P*_ara_*-flag-flhDC-HA construction |
| flhC_HA R | P-TAT GGG TAG CAT GCG GAT CCA ACA GCC TGT ACT CTC T | pCM-P*_ara_*-flag-flhDC-HA construction |
| fliA F | P-GTG AAT TCA CTC TAT ACC G | pCM-P*_ara_*-flag-fliA construction |
| fliA R | P-TTA TAA CTT ACC CAG TTT AGT GCG | pCM-P*_ara_*-flag-fliA construction |
| fliC F | P-ATG GCA CAA GTC ATT AAT AC | pCM-P*_ara_*-fliC construction |
| fliC R | P-TTA ACC CTG CAG CAG AG | pCM-P*_ara_*-fliC construction |
| GFP F | P-ATG AGT AAA GGA GAA GAA C | pT25-GFP construction |
| GFP R | P-TTA TTT GTA TAG TTC ATC C | pT25-GFP construction |
| flgM pro F | P-GGA ATG TGT TGC GTT GAG GTC | pT25-pflgM-GFP construction |
| flgM pro 30 R | P-CGC CCG GCT GTT CGT TA | pT25-pflgM-GFP construction |
| fliF pro F | P-CGG TTG CGG CAG TGA TT | pT25-pfliF-GFP construction |
| fliF pro 30 R | P-GGA ACC GGC AAC AAT CA | pT25-pfliF-GFP construction |
| fliM pro F | P-CGC ACC TGT TTT GAG T | pT25-pfliM-GFP construction |
| fliM 30 R | GCT AAC ACT GGC TGT C | pT25-pfliM-GFP construction |
| fliI pro F | P-TTA CTG ATA CGC CGC CAC | pT25-pfliI-GFP construction |
| fliI 26 R | ACG TAC CGC AGG CAA C | pT25-pfliI-GFP construction |
| flhA pro F | P-GGT TTG CCG ATC CGA TTG AC | pT25-pflhB-GFP construction |
| flhB 30 R | ACG GGA ACG CGG GAT | pT25-pflhB-GFP construction |
| fliF F | P-ATG AAT GCG ACT GCA GCC C | pFliF-Bs1 construction |
| fliF R | P-CTC ATG ATC GTT ATT TAT CCA CTG G | pFliF-Bs1 construction |
| Linker F | P-CTC GCT GAG GCC GCC | pFliF-Bs1 construction |
| Bs1 R | P-TCA CAT AAT CGG AAG CAC | pFliF-Bs1 construction |
| RNE_genome_200 FW | CAG TCC TTA CCG GTA GAT GG | *rne* gene sequencing |
| RNE down 360 | GAG TCG CTG GAT TCA TTA GG | *rne* gene sequencing |
| RNE FW | ATG AAA AGA ATG TTA ATC AAC GC | *rne* gene sequencing |
| RNE 550 | GCG CTG CAA TGG GAT | *rne* gene sequencing |
| RNE +1K seq | TGC GCC TGC GTG ACC TCG | *rne* gene sequencing |
| RNE 1750 | TGT TCA GCG GTG GTG | *rne* gene sequencing |
| RNE +2K seq | TAC CGC CGA CGA GCA GCA | *rne* gene sequencing |
| RNE 2650 | ACC GGT TGT TAG CGC | *rne* gene sequencing |
| RNE +3K seq | GTT GAG CAT AAC CAC GCT | *rne* gene sequencing |
| flh pro F3 | CAG CGA CAA GAA TAT TGC TAT AGC | *flhDC* promoter sequencing |
| flh pro R2 | CGG GAA TCT TGC GTC AAC TGA G | *flhDC* promoter sequencing |
| oxyS seq F | AAC ATC ACG CCC AGC TCA TC | oxyS gene sequencing |
| oxyS seq R | CAA ACC GCT GGA AGA TCT GC | oxyS gene sequencing |
| arcZ seq F | CAT CAA CCT GCT GCT TAT CC | arcZ gene sequencing |
| arcZ seq R | ACG GGT TAG CGG TGT TTG AG | arcZ gene sequencing |
| omrB seq F | GGA AAC GGT GGC GAC TGA AA | omrB gene sequencing |
| omrB seq R | GGG AAA TGC AAC GAG GTG TG | omrB gene sequencing |
| omrA seq F | CAT TTC CCT TCA TTC CTT TG | omrA gene sequencing |
| omrA seq R | ATG GAG GGA GAT TAC ACG AG | omrA gene sequencing |
| pBAD Fw | ATG CCA TAG CAT TTT TAT CC | pBAD plasmid sequencing |
| pBAD RW | GTT CAA ATC CGC TCC CG | pBAD plasmid sequencing |
| pcml128 seq R | GCA AGG AAA CTA CCC ATA ATA C | pCM128 plasmid sequencing |
| pcml128 seq F | CCC GAA AAG TGC CAC CTG AC | pCM128 plasmid sequencing |
| T25 seq F | GCC CCA TAC GAT ATA | pT25 plasmid sequencing |
| pT25-RW-seq | GTC ATA AGT GCG GCG ACG A | pT25 plasmid sequencing |
| fliF seq 1 | AAT CTT TCC GAT CAG | *fliF* gene sequencing |
| fliF seq 2 | AAG CGG TTC CGG TTA | *fliF* gene sequencing |
| fliI seq 1 | GAC CAT TGG CGA AGA | *fliI* gene sequencing |
| fliI seq 2 | ATT CTG CGC GAC GAT | *fliI* gene sequencing |
| fliI seq 3 | CAG GAG TGG TGT AAT GAC | *fliI* gene sequencing |
| fliI seq 4 | GTG TTG CTG ATT ATG GAC | *fliI* gene sequencing |
| fliM seq 1 | CCG CTG ACG ACA AAG | *fliM* gene sequencing |
| fliM seq 2 | GAA CAG CGC GTC ATC | *fliM* gene sequencing |
| flhA seq 1 | AAC AGA AGC CCC CAC | *flhBA* gene sequencing |
| flhA seq 2 | AAT AAC CCG ACC CAC | *flhBA* gene sequencing |
| flhA seq 3 | GGT CAT TAC CAA AGG | *flhBA* gene sequencing |
| flhA seq 4 | GGA TTT CTG CCG CCA | *flhBA* gene sequencing |

*Underline: homologous sequence for chromosomal fusion

P-: 5’ end phosphate

Table S3. Primers for oligo probes

| **Primer Name** | **Sequence (5' – 3')** | **Purpose** |
| --- | --- | --- |
| RNAI oligo | GTA ACT GGC TTC AGC AGA GCG CAG ATA CC | Northern blot |
| oxyS oligo | GTT CAC GTT GGC TTT AGT TAT TCG AGT TGA G | Northern Blot |
| mcaS oligo | CCA GAC TCT ACA GTA CAC ACA GCA GTG CAT CC | Northern Blot |
| arcZ oligo | CTT GGC TGC GCC GTA AAT TAT TAT GAT GAG TTA C | Northern Blot |
| omrA oligo | AGG TTG GTG CAA GAG ACA GGG TAC GAA GAG CGT ACC G | Northern Blot |
| omrB oligo | GCA GGC TGG TGT AAT TCA TGT GCT CAA CCC GAA GTT GA | Northern Blot |

Table S4. Primers for qRT-PCR

| **Primer name** | **Sequence (5' – 3')** | **Amplification Fragment (bp)** | **Reference** |
| --- | --- | --- | --- |
| flgK qF | AGC TTC ACG CTG AAA CCA GT | 161 | Fan *et al.* |
| flgK qR | CCC ACC GTT TTA CTG TTG CT |  | Fan *et al.* |
| fliA qF | CGA ACG CTA TGA CGC CCT AC | 150 | Fan *et al.* |
| fliA qR | TGC CCT ATT GCC TGT GCC |  | Fan *et al.* |
| flgB qF | GTG GTT GCA CTG ACG ATG AC | 159 | Fan *et al.* |
| flgB qR | CAG GCT GTT ATC GGC AAA CT |  | Fan *et al.* |
| fliF qF | TCC TGT CGC CTA TTG TTG G | 94 | Fan *et al.* |
| fliF qR | CGA TAC TGT TCT TCC GTT TGT |  | Fan *et al.* |
| flgA qF | CAG CGG TGA TGG GTT TAG | 123 | Fitzgerald *et al*. |
| flgA qR | CCA TCT GCA TCA ACA ACG |  | Fitzgerald *et al*. |
| flgM qF | CAG CAC CAG TGT GAC GTT | 97 | Fitzgerald *et al*. |
| flgM qR | CGC CAG TTT TAA CGC TTC |  | Fitzgerald *et al*. |
| flhB qF | ATG CGG AGA TTG GTC AAC | 136 | Fitzgerald *et al*. |
| flhB qR | GGC ACC GGA AGA TGA GTA |  | Fitzgerald *et al*. |
| fliD qF | CAG CAA CGG TAT GGA GGT | 136 | Fitzgerald *et al*. |
| fliD qR | CGT GGT GAC ATC GTT CAG |  | Fitzgerald *et al*. |
| fliE qF | AGG GGA TTG AAG GGG TTA | 94 | Fitzgerald *et al*. |
| fliE qR | CCG GCA AAA CTA ATG GTC |  | Fitzgerald *et al*. |
| fliL qF | AAG TCC GTA GTC GCT TGC | 142 | Fitzgerald *et al*. |
| fliL qR | ACA TCC TGT TTC GGT TGC |  | Fitzgerald *et al*. |
| 16s qF | CAC AAG CGG TGG AGC AT | 198 | Fan *et al.* |
| 16s qR | CTG GCA ACA AAG GAT AAG G |  | Fan *et al.* |
| mreB qF | CGG TTC TAT GGT GGT TGA | 88 | Fitzgerald *et al*. |
| mreB qR | GCG CAC AGA AGA GGA GTA |  | Fitzgerald *et al*. |
| flhD qF | CTT GCA CAG CGT TTG ATT GT | 225 | Fan *et al.* |
| flhD qR | GAT GCC GGT ATG AAT TTG CT |  | Fan *et al.* |
| flhC qF | CCC ACA AGC AGA AGA AGG | 173 | Fan *et al.* |
| flhC qR | ATG GCG GTT GAC ATA AGC |  | Fan *et al.* |
| fliC qF | TTT GAC TGC TGG TGG TGA | 136 | Fitzgerald *et al*. |
| fliC qR | ATC CAG ACG GTT TTG CAC |  | Fitzgerald *et al*. |
| motA qF | CTC TAT CAA CCC GCT GAA CT | 141 | Fitzgerald *et al*. |
| motA qR | CAT TGC TTT GGT GTA TTT GG |  | Fitzgerald *et al*. |
| tar qF | CTT ATT TCC GGC AGT CTG TT | 115 | Fitzgerald *et al*. |
| tar qR | GCA TTA AAT CCC AGG TTG AC |  | Fitzgerald *et al*. |
| cheR qF | CGT TCT GGC TGA CCA TAA ACG CG | 194 | Partridge *et al*. |
| cheR qR | GAG GGA AAT GAT GTG CCT CAC GG |  | Partridge *et al*. |
| cheA qF | CAG CTC GAC GCT TAT AAA CAG TCG C | 189 | Partridge *et al*. |
| cheA qR | GAT AAT TCG TCG CGG CGA CTG AC |  | Partridge *et al*. |
| cheY qF | GTG CGT AAC CTG CTG AAA GAG CTG | 183 | Partridge *et al*. |
| cheY qR | CAA TGC CGA CAT CGC GCC ATC |  | Partridge *et al*. |
| fliI qF | GAG TGC GCA CCT TCA AAC AG | 112 | This study |
| fliI qR | AGG GCG ATG GCT TTA TCG AG |  | This study |
| fliQ qF | GAT GGG GAC TGA AGC GAT GA | 59 | This study |
| fliQ qR | CTA CCA ACA ATA GCG GGG CA |  | This study |
| flgI qF | GTA GCG CAG GGG AAT CTC TC | 79 | This study |
| flgI qR | GTC CAC CAC CAA ACG GTG TA |  | This study |
| fliG qF | CGC AGG TGG AAA ACG AAC AG | 96 | This study |
| fliG qR | CAT AGG TAT CCT CGC CGC TG |  | This study |
| fliH qF | GCT GTA AAG TCT CCG CCG AT | 67 | This study |
| fliH qR | CAG AGT TCT TGC CAG CGA GT |  | This study |
| fliJ qF | AGC AAC CGC TGG ATC AAC TA | 71 | This study |
| fliJ qR | TGC TGG CGA TGC TGA GTA AT |  | This study |
| fliK qF | GCC CAG AGT AAA GCG GAA GT | 144 | This study |
| fliK qR | TTG CCA TTC GTG AGA ACC CA |  | This study |
| flhA qF | TGG CGA TTA ACC CTG GAA CC | 138 | This study |
| flhA qR | CCT CAA CCA CTG TGT ACC CC |  | This study |
| flhE qF | TGG TAG CGT GGC GTT ATC AG | 100 | This study |
| flhE qR | CCG CTC TGC CCC TCT AAT TC |  | This study |
| fliZ qF | CCA GCA CCA ACA ATT ACC GC | 97 | This study |
| fliZ qR | GAA GGC AGG CTG GAG GAT TT |  | This study |
| fliY qF | ATC CTC GTT GAT CGT CTG GC | 66 | This study |
| fliY qR | GGT TAC TGC CAG CGT ATC GT |  | This study |
| flgN qF | AAC GGC TGG TTA CTG GAA GG | 117 | This study |
| flgN qR | GCG ATG GGT TGT TGA GGT CT |  | This study |
| flgC qF | GCG AAT GCT GAT AGC GTG AC | 117 | This study |
| flgC qR | AAC ATC GGC AAC CTT TAC GC |  | This study |
| flgD qF | TAC CAC CAG CAG TAG TTC GC | 104 | This study |
| flgD qR | GAT TGG TCG GGT CCT GGT TT |  | This study |
| flgE qF | GAA TAC CCT GAT GGC AGC GA | 88 | This study |
| flgE qR | TGG CGT AAC AGT AGG AAG CG |  | This study |
| flgF qF | GGT GAC GAC GGC ATT TTT CG | 103 | This study |
| flgF qR | CTT CCA GAA CCC CCG ACA TC |  | This study |
| flgG qF | GTC AGC GTA ACC CAA CAA GG | 126 | This study |
| flgG qR | AGA GGA TTG CGT TTC GGT GT |  | This study |
| flgH qF | GCA ATA CCT TTA GCG GCA CG | 129 | This study |
| flgH qR | CCA CGC CAG AGA AGC GAA TA |  | This study |
| flgJ qF | CGA ACT AAA GGC GAA AGC GG | 184 | This study |
| flgJ qR | TTG GGC AAT CTG CTG GTC AT |  | This study |
| flgL qF | CCA GGT CAC CAC TGC TAT CC | 75 | This study |
| flgL qR | TCA TCG TCA CTC AAG GTG CC |  | This study |
| fliS qF | ACT GCG GGT GAG TCT TGA TG | 168 | This study |
| fliS qR | TCT TTC CAG GCA TCG GCA AT |  | This study |
| fliT qF | ATT TCG CCT GGC AAC AAC TC | 149 | This study |
| fliT qR | GGA CGG GTC AAC CTC TTC AG |  | This study |
| fliM qF | AGT GTT AGC GGC GAA AGT GA | 134 | This study |
| fliM qR | AGG TTG AAC AGC CCC ATA CG |  | This study |
| fliN qF | CAA TGG ACG ATC TGT GGG CT | 60 | This study |
| fliN qR | CGC TTT TGC TGC TGG TTG AT |  | This study |
| fliO qF | GGC TGG TAA AAC GGT TGG GA | 105 | This study |
| fliO qR | CGA CCA CCA CAA CCC TTT C |  | This study |
| fliP qF | ACC AGT TTC ACC CGC ATC AT | 175 | This study |
| fliP qR | TCT CTT CGC TGA ATG GCT GG |  | This study |
| fliR qF | ACG GGT AAA ACT GGG TCT G | 101 | This study |
| fliR qR | CCA GCC ACA GAG CAA AGA A |  | This study |
| tap qF | TGC TGC GGG TAA TCT GGC G | 116 | This study |
| tap qR | GCA CAT CAC TTA CCG TCC CA |  | This study |
| cheB qF | CGG ATG GAC GGA CTG GAT TT | 102 | This study |
| cheB qR | CAG CGT GAC TTC TGA CCC TT |  | This study |
| cheZ qF | GTT TGC CGA TCC GAT TGA CC | 116 | This study |
| cheZ qR | TGA TTT CCA GCA GTT GCG |  | This study |
| motB qF | TGG TCC AGG AAG GTC TAC G | 180 | This study |
| motB qR | TGG CGT AGG GGA AAT CAT C |  | This study |
| cheW qF | TCA GGT AAC ACG GAT TGC | 152 | This study |
| cheW qR | CGA GAT TCA GGA CGA TAA CTA |  | This study |

Table S5. Complete qRT-PCR raw data of Figure 4B.

|  | MG1655 | | | | Rned500 | | | |  |
| --- | --- | --- | --- | --- | --- | --- | --- | --- | --- |
| target | relative expression | relative expression (log2) | S.D. | n | relative expression | relative expression (log2) | S.D. | n | p-value |
| flhD | 1.00 | 0.01 | 0.12 | 3 | 1.87 | 0.91 | 0.39 | 3 | 0.011 |
| flhC | 1.00 | 0.00 | 0.07 | 3 | 2.39 | 1.26 | 0.32 | 3 | 0.001 |
| flhB | 1.04 | 0.06 | 0.36 | 3 | 11.34 | 3.50 | 3.30 | 3 | 0.001 |
| flhA | 1.01 | 0.02 | 0.21 | 3 | 1.38 | 0.46 | 0.19 | 3 | 0.095 |
| flhE | 1.03 | 0.04 | 0.29 | 3 | 5.14 | 2.36 | 0.71 | 3 | 0.001 |
| fliE | 1.01 | 0.01 | 0.15 | 3 | 2.87 | 1.52 | 1.12 | 3 | 0.020 |
| fliF | 1.03 | 0.04 | 0.30 | 3 | 23.12 | 4.53 | 8.17 | 3 | 0.000 |
| fliG | 1.05 | 0.07 | 0.45 | 3 | 5.43 | 2.44 | 2.07 | 3 | 0.020 |
| fliH | 1.04 | 0.06 | 0.35 | 3 | 5.82 | 2.54 | 1.89 | 3 | 0.003 |
| fliI | 1.02 | 0.02 | 0.26 | 3 | 3.28 | 1.71 | 1.02 | 3 | 0.024 |
| fliJ | 1.08 | 0.11 | 0.48 | 3 | 3.94 | 1.98 | 1.28 | 3 | 0.016 |
| fliK | 1.05 | 0.08 | 0.41 | 3 | 3.34 | 1.74 | 1.08 | 3 | 0.016 |
| flgA | 1.02 | 0.03 | 0.26 | 3 | 5.64 | 2.50 | 1.36 | 3 | 0.001 |
| flgB | 1.04 | 0.06 | 0.33 | 3 | 2.83 | 1.50 | 0.45 | 3 | 0.010 |
| flgC | 1.07 | 0.10 | 0.48 | 3 | 2.77 | 1.47 | 0.58 | 3 | 0.025 |
| flgD | 1.06 | 0.09 | 0.45 | 3 | 1.79 | 0.84 | 0.52 | 3 | 0.137 |
| flgE | 1.06 | 0.09 | 0.45 | 3 | 1.73 | 0.79 | 0.44 | 3 | 0.135 |
| flgF | 1.07 | 0.10 | 0.46 | 3 | 2.36 | 1.24 | 0.52 | 3 | 0.044 |
| flgG | 1.05 | 0.07 | 0.38 | 3 | 2.49 | 1.31 | 0.89 | 3 | 0.044 |
| flgH | 1.04 | 0.06 | 0.33 | 3 | 3.92 | 1.97 | 1.35 | 3 | 0.009 |
| flgI | 1.03 | 0.05 | 0.31 | 3 | 4.40 | 2.14 | 1.11 | 3 | 0.004 |
| flgJ | 1.03 | 0.05 | 0.32 | 3 | 4.75 | 2.25 | 1.37 | 3 | 0.003 |
| fliA | 1.08 | 0.11 | 0.48 | 3 | 4.48 | 2.16 | 1.26 | 3 | 0.012 |
| fliZ | 1.05 | 0.07 | 0.40 | 3 | 2.67 | 1.42 | 0.75 | 3 | 0.027 |
| fliY | 1.04 | 0.06 | 0.36 | 3 | 0.71 | -0.49 | 0.30 | 3 | 0.271 |
| fliD | 1.01 | 0.02 | 0.23 | 3 | 0.61 | -0.72 | 0.18 | 3 | 0.092 |
| fliS | 1.03 | 0.04 | 0.35 | 3 | 0.52 | -0.93 | 0.25 | 3 | 0.156 |
| fliT | 1.02 | 0.03 | 0.29 | 3 | 0.74 | -0.43 | 0.28 | 3 | 0.342 |
| fliL | 1.03 | 0.04 | 0.30 | 3 | 5.34 | 2.42 | 2.04 | 3 | 0.005 |
| fliM | 1.04 | 0.06 | 0.35 | 3 | 8.39 | 3.07 | 2.71 | 3 | 0.002 |
| fliN | 1.07 | 0.09 | 0.44 | 3 | 6.70 | 2.74 | 2.37 | 3 | 0.005 |
| fliO | 1.07 | 0.09 | 0.47 | 3 | 15.05 | 3.91 | 6.88 | 3 | 0.002 |
| fliP | 1.04 | 0.06 | 0.35 | 3 | 19.21 | 4.26 | 9.53 | 3 | 0.002 |
| fliQ | 1.01 | 0.02 | 0.21 | 3 | 9.65 | 3.27 | 3.35 | 3 | 0.001 |
| fliR | 1.01 | 0.02 | 0.22 | 3 | 5.37 | 2.42 | 2.98 | 3 | 0.008 |
| flgK | 1.10 | 0.14 | 0.53 | 3 | 1.25 | 0.32 | 0.28 | 3 | 0.603 |
| flgL | 1.02 | 0.03 | 0.31 | 3 | 1.08 | 0.11 | 0.21 | 3 | 0.781 |
| flgM | 1.04 | 0.06 | 0.35 | 3 | 1.00 | -0.01 | 0.25 | 3 | 0.924 |
| flgN | 1.04 | 0.05 | 0.35 | 3 | 2.01 | 1.01 | 0.35 | 3 | 0.031 |
| fliC | 1.04 | 0.06 | 0.42 | 3 | 0.41 | -1.27 | 0.05 | 3 | 0.034 |
| motA | 1.01 | 0.02 | 0.23 | 3 | 0.24 | -2.06 | 0.05 | 3 | 0.006 |
| motB | 1.01 | 0.02 | 0.21 | 3 | 0.37 | -1.42 | 0.08 | 3 | 0.013 |
| cheA | 1.02 | 0.03 | 0.27 | 3 | 0.09 | -3.40 | 0.01 | 3 | 0.001 |
| cheW | 1.02 | 0.03 | 0.30 | 3 | 0.22 | -2.20 | 0.03 | 3 | 0.004 |
| tar | 1.02 | 0.03 | 0.27 | 3 | 0.07 | -3.89 | 0.01 | 3 | 0.001 |
| tap | 1.03 | 0.05 | 0.36 | 3 | 0.06 | -3.96 | 0.01 | 3 | 0.001 |
| cheR | 1.01 | 0.02 | 0.23 | 3 | 0.12 | -3.01 | 0.02 | 3 | 0.001 |
| cheB | 1.03 | 0.04 | 0.35 | 3 | 0.10 | -3.28 | 0.02 | 3 | 0.002 |
| cheY | 1.03 | 0.04 | 0.33 | 3 | 0.13 | -2.98 | 0.01 | 3 | 0.002 |
| cheZ | 1.02 | 0.03 | 0.29 | 3 | 0.26 | -1.95 | 0.03 | 3 | 0.004 |

**SI References**

1. C. S. Barker, B. M. Pruss, P. Matsumura, Increased motility of Escherichia coli by insertion sequence element integration into the regulatory region of the flhD operon. *Journal of bacteriology* **186**, 7529-7537 (2004).

2. K. A. Fahrner, H. C. Berg, Mutations That Stimulate flhDC Expression in Escherichia coli K-12. *Journal of bacteriology* **197**, 3087-3096 (2015).

3. K. A. Datsenko, B. L. Wanner, One-step inactivation of chromosomal genes in Escherichia coli K-12 using PCR products. *Proceedings of the National Academy of Sciences of the United States of America* **97**, 6640-6645 (2000).

4. S. Nurmohamed, A. R. McKay, C. V. Robinson, B. F. Luisi, Molecular recognition between Escherichia coli enolase and ribonuclease E. *Acta crystallographica. Section D, Biological crystallography* **66**, 1036-1040 (2010).

5. O. N. Murashko, S. Lin-Chao, Escherichia coli responds to environmental changes using enolasic degradosomes and stabilized DicF sRNA to alter cellular morphology. *Proceedings of the National Academy of Sciences of the United States of America* **114**, E8025-E8034 (2017).

6. L. C. Thomason, N. Costantino, D. L. Court, E. coli genome manipulation by P1 transduction. *Curr Protoc Mol Biol* **Chapter 1**, 1 17 11-11 17 18 (2007).

7. T. Baba *et al.*, Construction of Escherichia coli K-12 in-frame, single-gene knockout mutants: the Keio collection. *Mol Syst Biol* **2**, 2006 0008 (2006).

8. M. Tamura *et al.*, RNase E maintenance of proper FtsZ/FtsA ratio required for nonfilamentous growth of Escherichia coli cells but not for colony-forming ability. *Journal of bacteriology* **188**, 5145-5152 (2006).

9. W. T. Tucker, C. A. Miller, S. N. Cohen, Structural and functional analysis of the par region of the pSC 10 1 plasmid. *Cell* **38**, 191-201 (1984).

10. G. Karimova, J. Pidoux, A. Ullmann, D. Ladant, A bacterial two-hybrid system based on a reconstituted signal transduction pathway. *Proceedings of the National Academy of Sciences of the United States of America* **95**, 5752-5756 (1998).

11. H. Liu, J. H. Naismith, An efficient one-step site-directed deletion, insertion, single and multiple-site plasmid mutagenesis protocol. *BMC Biotechnol* **8**, 91 (2008).

12. A. J. Callaghan *et al.*, Structure of Escherichia coli RNase E catalytic domain and implications for RNA turnover. *Nature* **437**, 1187-1191 (2005).

13. C. Jain, J. G. Belasco, RNase E autoregulates its synthesis by controlling the degradation rate of its own mRNA in Escherichia coli: unusual sensitivity of the rne transcript to RNase E activity. *Genes Dev* **9**, 84-96 (1995).

14. S. Sousa, I. Marchand, M. Dreyfus, Autoregulation allows Escherichia coli RNase E to adjust continuously its synthesis to that of its substrates. *Mol Microbiol* **42**, 867-878 (2001).

15. T. Drepper *et al.*, Reporter proteins for in vivo fluorescence without oxygen. *Nature Biotechnology* **25**, 443-445 (2007).

16. K. Maeda, Y. Imae, J. I. Shioi, F. Oosawa, Effect of temperature on motility and chemotaxis of Escherichia coli. *Journal of bacteriology* **127**, 1039-1046 (1976).

17. J. Schindelin *et al.*, Fiji: an open-source platform for biological-image analysis. *Nat Methods* **9**, 676-682 (2012).

18. C. Chen, M. P. Deutscher, RNase R is a highly unstable protein regulated by growth phase and stress. *RNA* **16**, 667-672 (2010).

19. C. L. Wisseman, Jr., J. E. Smadel, F. E. Hahn, H. E. Hopps, Mode of action of chloramphenicol I: action of chloramphenicol on assimilation of ammonia and on synthesis of proteins and nucleic acids in Escherichia coli. *Journal of bacteriology* **67**, 662-673 (1954).

20. J. E. Karlinsey *et al.*, Completion of the hook-basal body complex of the Salmonella typhimurium flagellum is coupled to FlgM secretion and fliC transcription. *Mol Microbiol* **37**, 1220-1231 (2000).

21. S. Lin-Chao, S. N. Cohen, The rate of processing and degradation of antisense RNAI regulates the replication of ColE1-type plasmids in vivo. *Cell* **65**, 1233-1242 (1991).

22. W. Schulz, W. Zillig, Rifampicin inhibition of RNA synthesis by destabilisation of DNA-RNA polymerase-oligonucleotide-complexes. *Nucleic Acids Res* **9**, 6889-6906 (1981).

23. L. Jiang *et al.*, Synthetic spike-in standards for RNA-seq experiments. *Genome Res* **21**, 1543-1551 (2011).

24. A. Dobin *et al.*, STAR: ultrafast universal RNA-seq aligner. *Bioinformatics* **29**, 15-21 (2013).

25. B. Li, C. N. Dewey, RSEM: accurate transcript quantification from RNA-Seq data with or without a reference genome. *BMC Bioinformatics* **12**, 323 (2011).

26. D. M. Fitzgerald, R. P. Bonocora, J. T. Wade, Comprehensive mapping of the Escherichia coli flagellar regulatory network. *PLoS genetics* **10**, e1004649 (2014).

27. K. J. Livak, T. D. Schmittgen, Analysis of relative gene expression data using real-time quantitative PCR and the 2(-Delta Delta C(T)) Method. *Methods* **25**, 402-408 (2001).

28. M. K. Thomason, F. Fontaine, N. De Lay, G. Storz, A small RNA that regulates motility and biofilm formation in response to changes in nutrient availability in Escherichia coli. *Mol Microbiol* **84**, 17-35 (2012).

29. X. Li *et al.*, Helicobacter pylori induces IL-1β and IL-18 production in human monocytic cell line through activation of NLRP3 inflammasome via ROS signaling pathway. *Pathogens and disease* **73**, ftu024 (2015).

30. A. K. Dickey *et al.*, Flagellin-independent effects of a Toll-like receptor 5 polymorphism in the inflammatory response to Burkholderia pseudomallei. *PLoS Negl Trop Dis* **13**, e0007354 (2019).

31. F. Mercurio, J. A. DiDonato, C. Rosette, M. Karin, p105 and p98 precursor proteins play an active role in NF-kappa B-mediated signal transduction. *Genes Dev* **7**, 705-718 (1993).

32. M. Noursadeghi *et al.*, Quantitative imaging assay for NF-κB nuclear translocation in primary human macrophages. *Journal of immunological methods* **329**, 194-200 (2008).

33. U. Schindler, V. R. Baichwal, Three NF-kappa B binding sites in the human E-selectin gene required for maximal tumor necrosis factor alpha-induced expression. *Mol Cell Biol* **14**, 5820-5831 (1994).

34. D. Zipser, L. Lipsich, J. Kwoh, Mapping functional domains in the promoter region of the herpes thymidine kinase gene. *Proceedings of the National Academy of Sciences* **78**, 6276-6280 (1981).

35. G. Grentzmann, J. A. Ingram, P. J. Kelly, R. F. Gestaland, J. F. Atkins, A dual-luciferase reporter system for studying recoding signals. *RNA* **4**, 479-486 (1998).

36. T. L. Bailey, N. Williams, C. Misleh, W. W. Li, MEME: discovering and analyzing DNA and protein sequence motifs. *Nucleic acids research* **34**, W369-W373 (2006).

37. G. A. Mackie, RNase E: at the interface of bacterial RNA processing and decay. *Nat Rev Microbiol* **11**, 45-57 (2013).

38. F. R. Blattner *et al.*, The complete genome sequence of Escherichia coli K-12. *Science* **277**, 1453-1462 (1997).

39. C. H. Chang *et al.*, TBK1-associated protein in endolysosomes (TAPE) is an innate immune regulator modulating the TLR3 and TLR4 signaling pathways. *J Biol Chem* **286**, 7043-7051 (2011).

40. Y. Fan, C. Evans, J. Ling (2016) Reduced protein synthesis fidelity inhibits flagellar biosynthesis and motility. Sci Rep 6: 30960.

41. J. D. Partridge, N. T. Q. Nhu, Y. S. Dufour, R. M. Harshey, Escherichia coli Remodels the Chemotaxis Pathway for Swarming. *mBio* **10**, e00316-00319 (2019).
